# Assembling giant heterometallic cyclic coordination clusters {Mn^II^_2_Ln^III^_2_}_*n*_ (*n* = 6 and 8) for efficient photocatalytic hydrogen production

**DOI:** 10.1039/d6sc06444b

**Published:** 2026-09-15

**Authors:** Kai-Peng Bai, Yu Wang, Wei-Peng Chen, Yuan-Qi Zhai, Qian-Cheng Luo, Xiang-Quan Hu, Yan-Zhen Zheng

**Affiliations:** a Frontier Institute of Science and Technology, Interdisciplinary Research Center of Frontier science and technology, Xi'an Key Laboratory of Electronic Devices and Materials Chemistry, School of Future Technology, College of Forensic Medicine, School of Chemistry, Xi'an Jiaotong University Xi'an 710054 China zheng.yanzhen@xjtu.edu.cn; b School of Materials Science and Engineering, Shaanxi University of Technology Hanzhong 723001 China sxchenweipeng@163.com

## Abstract

Heterometallic Mn–rare earth metal clusters with exceptional catalytic activity remain scarcely explored. Herein, two nanosized oxo/alkoxo-bridged cyclic coordination clusters (CCCs), namely {Mn_12_Ln_12_} and {Mn_16_Ln_16_}, were successfully isolated. These elegant supramolecular aggregates represent rare examples of Mn(ii)-containing 3d–4f clusters, as Mn(ii) ions are highly susceptible to aerial oxidation under alkaline conditions. Notably, {Mn_16_Ln_16_} constitutes the largest such cluster reported to date in terms of nuclearity. Interestingly, both CCCs are composed of repetitive {Mn_2_Ln_2_O_4_} structural units, and their structures have certain similarities to some characteristics of the cubic {CaMn_4_O_5_} oxygen-evolving center in photosystem II. Crucially, these clusters exhibit excellent stability in solvents and function as highly efficient homogeneous photocatalysts for H_2_ production. Specifically, {Mn_12_Gd_12_} achieves an outstanding photocatalytic hydrogen production rate of 2778 µmol g^−1^ h^−1^, surpassing that of many reported covalent organic frameworks (COFs) and precious-metal-free inorganic photocatalysts. This superior catalytic performance stems from its suitable energy levels, high charge-transfer efficiency, and abundance of active oxygen sites. This work offers new mechanistic insights and design principles for the rational development of highly active manganese-cluster-based photocatalysts.

## Abstract 2

Two series of Mn–Ln nanoclusters were synthesized, the {Mn_12_Gd_12_} cluster shows high activity for photocatalytic hydrogen production due to its suitable energy levels high charge transfer efficiency and abundant reactive oxygen species sites.

## Graphical abstract

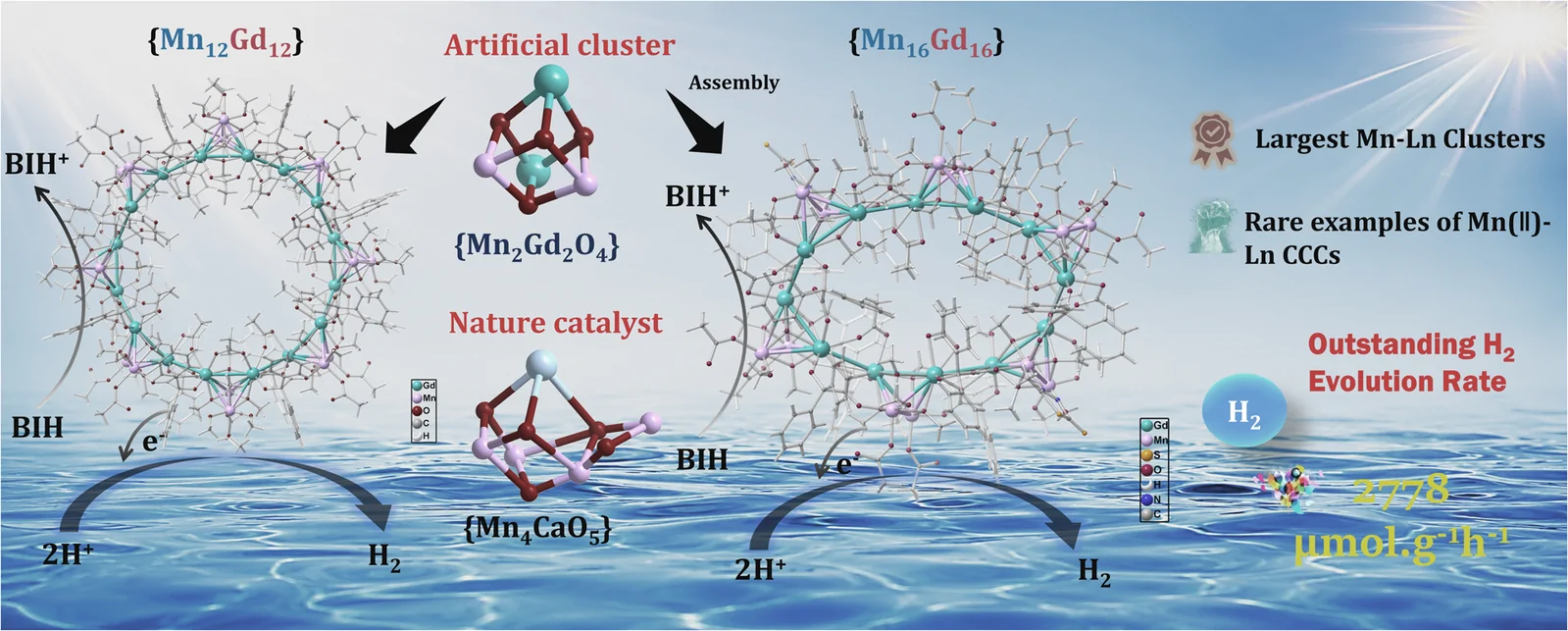

## Introduction

Converting solar energy into chemical energy is widely regarded as one of the promising and sustainable strategies for addressing environmental challenges and alleviating the global energy crisis, with the development of highly efficient and robust photocatalysts constituting its scientific and technological cornerstone.^1–3^ Recently, polymetallic nanoclusters characterized by atomic-level structural precision have shown exceptional potential in photocatalysis, particularly for CO_2_ reduction and water splitting, owing to their tunable light absorption, unique electronic structures, and abundant active sites.^4–10^ Among these, high-nuclearity 3d–4f clusters have also attracted considerable attention due to their rich diversity of metal-based active centers and the redox properties inherent to 3d and 4f metals.^11,12^ Furthermore, the synergistic interplay between 3d and 4f metal ions offers a versatile strategy for rationally modulating both the catalytic activity and product selectivity of such systems.^13–16^

However, reports on the catalytic applications of high-nuclearity 3d–4f heterometallic clusters remain exceedingly scarce.^17–20^ On one hand, 3d and 4f metal ions differ markedly in chemical affinity, coordination preferences, and configurational flexibility, which poses substantial challenges to the rational design and controlled assembly of such clusters, particularly high-nuclearity Mn–Ln systems.^21–23^ Manganese (Mn), as an essential redox-active center in natural photosynthesis, holds exceptional promise for multi-electron-transfer catalysis.^24–27^ Nevertheless, compared with Ni(ii) and Co(ii) ions, which exhibit stable oxidation states and well-defined coordination geometries and numbers, Mn(ii) is significantly more susceptible to undesired oxidative side reactions (*e.g.*, aerial or ligand-mediated oxidation to Mn(iii)/Mn(iv)) during cluster self-assembly. Moreover, its hydrolytic behavior is highly pH-sensitive, further complicating precise regulation of the coordination environment. Consequently, the vast majority of reported high-nuclearity 3d–4f clusters are based on Ni/Co–Ln systems, whereas structurally sophisticated and functionally promising Mn(ii)–Ln clusters remain exceptionally rare.^28–32^ On the other hand, such nanoscale clusters typically suffer from low solution-phase stability and poor reproducibility, which critically hinders reliable assessment of their intrinsic catalytic performance and impedes mechanistic investigation.^17,33,34^ Therefore, the development of Mn–Ln clusters combining high photocatalytic activity with robust solution stability remains a significant and unresolved challenge.

Herein, by controlling the dosage of organic base (Scheme S1), two high-nuclearity cyclic coordination clusters (CCCs), identified as [Mn^II^_12_Ln^III^_12_(µ_3_-OH)_2_(*µ*_3_-OCH_3_)_22_(dpga)_12_(mmt)_2_(CH_3_COO)_10_(CH_3_COO·H)_6_(CH_3_OH)_12_(H_2_O)_2_]·7H_2_O·5CH_3_CN·6CH_3_OH (Ln = Gd 1, Eu 2 and Tb 3) and [Mn^II^_16_Ln^III^_16_(µ_3_-OCH_3_)_32_(dpga)_16_(CH_3_COO)_16_(CH_3_COOH)_8_(CH_3_O H)_20_]·14H_2_O·5CH_3_OH·11CH_3_CN (Ln = Gd 4, Eu 5 and Tb 6), were successfully assembled from the solvothermal reactions of Mn(CH_3_COO)_2_·4H_2_O, Ln(NO_3_)·6H_2_O, and H_2_dpga and Hmmt ligands (H_2_dpga = diphenylglycolic acid and Hmmt = 2-mercapto-5-methyl-1,3,4-thiadiazole). Significantly, {Mn_16_Ln_16_} represents the largest Mn(ii)–Ln clusters discovered to date. The above clusters retain good structural stability in H_2_O/DMSO mixtures and show excellent photocatalytic H_2_ production. Notably, {Mn_12_Gd_12_} achieves a higher H_2_ evolution rate than many reported catalysts.

## Results and discussion

### Structure and solution stability

**Fig. 1:**
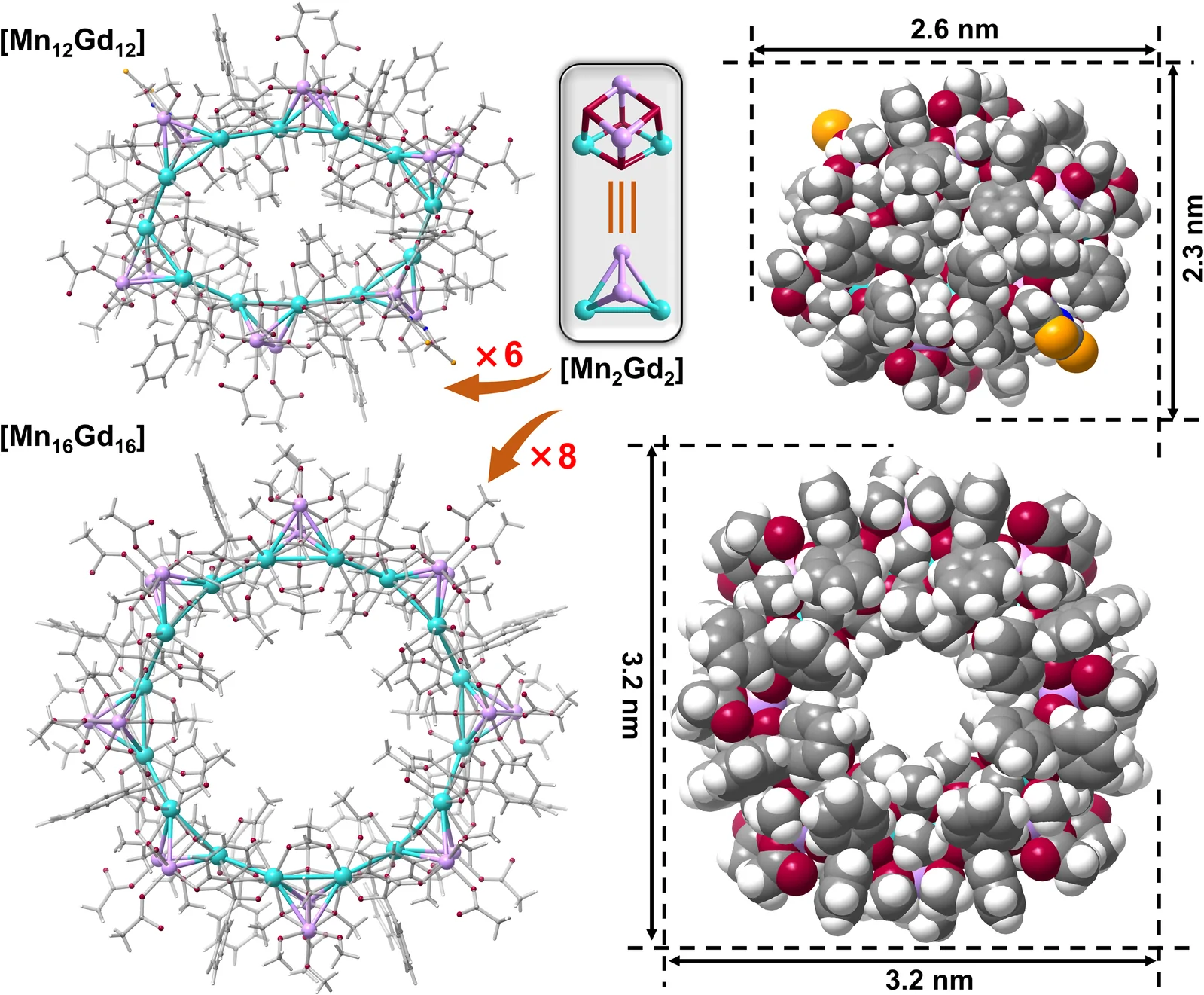
Ball-and-stick views and the space filling diagrams for 1 and 4. The metallic skeletons of {Mn_12_Gd_12_} and {Mn_16_Gd_16_} can be viewed as assembled from different amounts of {Mn_2_Gd_2_} units. Color code: Gd, cyan; Mn(ii), pink; S, yellow; N, blue; O, red; C, grey; H, white.

**Fig. 2:**
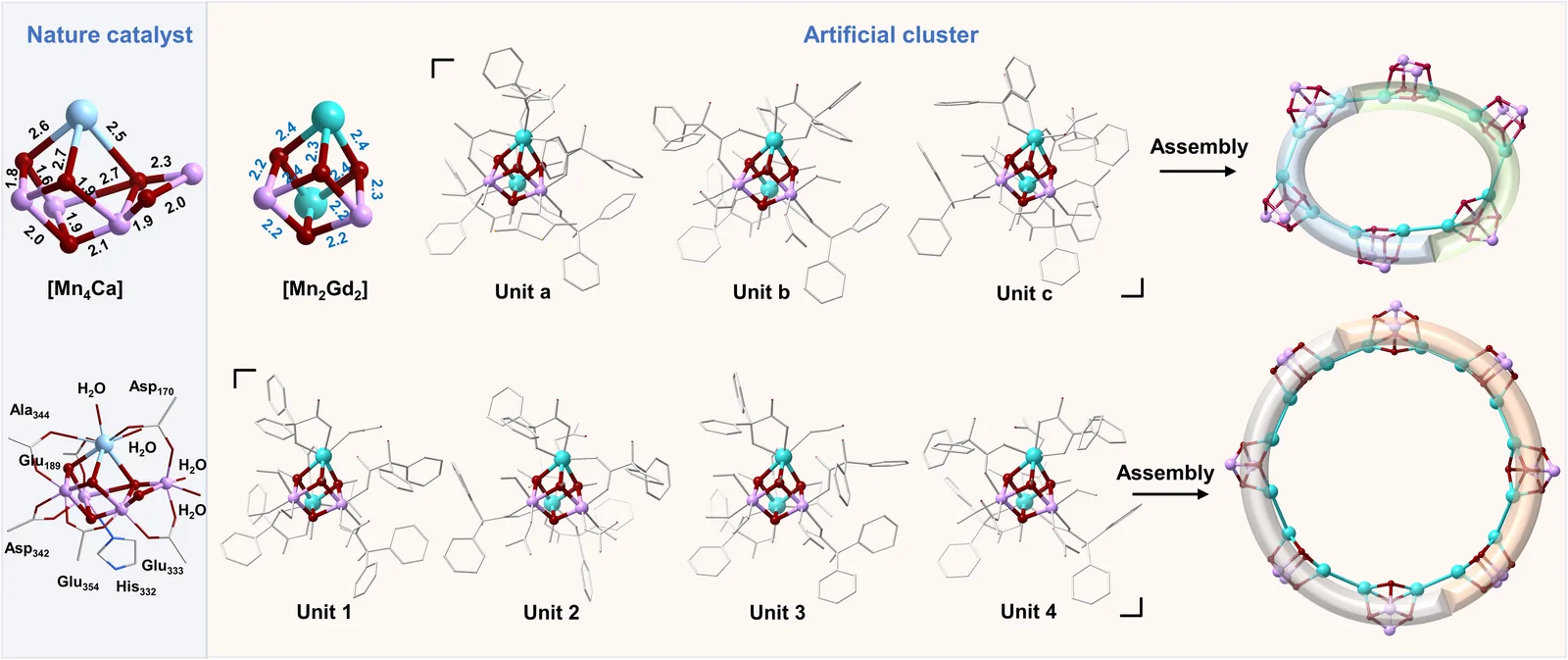
Crystal structures of the native {Mn_4_Ca} cluster and the structural building blocks of clusters 1 and 4 (cluster 1 consists of three building blocks: units a, b, and c; cluster 4 consists of four building blocks: units 1, 2, 3, and 4).

Single-crystal X-ray diffraction analysis reveals that 1 crystallizes in the triclinic space group *P*1̄ (Table S1). The oxidation states of the manganese centers in {Mn_12_Gd_12_} are corroborated by bond valence sum (BVS) calculations (Table S2). As illustrated in Fig. 1, the elliptical structure of 1 can be described as a {Mn_12_Gd_12_} torus composed of three kinds of [Mn_2_Gd_2_] sub-units (unit a, unit b and unit c) (Fig. 2). Interestingly, these [Mn_2_Gd_2_] subunits contain cubane-like metal-oxo motifs that are reminiscent of structural elements found in the oxygen–evolving complex of photosystem II (PSII).^24^ In the [Mn_2_Gd_2_] sub-unit structure, the distances of Mn–O and Gd–O bonds range from 2.17–2.30 Å and 2.33–2.41 Å, also very close to those of the Mn–O and Ca–O bonds in the cubic [Mn_4_Ca] (Table S8). For the first type of [Mn_2_Gd_2_] unit a, there is one mono-dentate deprotonated mmt^−^ ligand, one H_2_O and two CH_3_OH molecules, four µ_3_-OCH_3_ and one acetate distributed in the peripheries of the [Mn_2_Gd_2_] unit to satisfy the coordination requirement of metal ions and balance the charge. As shown in Fig. 2, the whole molecule 1 contains two such [Mn_2_Gd_2_] units. For the remaining two kinds of [Mn_2_Gd_2_] cubanes, there are some CH_3_OH and acetic acid molecules surrounding [Mn_2_Gd_2_(µ_3_-OCH_3_)_4_]. And then, six [Mn_2_Gd_2_] subunits were further linked together *via* twelve deprotonated dpga^2−^ ligands with 3.121 bridging modes (in Harris notation^35^) and six acetate anions adopting µ–η:^2^ η^1^ coordination modes to form the circular structure (Fig. S2).

Notarized by continuous-shape measurements (CShM; Tables S4 and S5),^36^ geometrical configurations for all Mn(ii) ions are in octahedral geometries and Gd(iii) centres show two geometric configurations (triangular dodecahedron and a biaugmented trigonal prism). The Gd–O and Mn–O bond lengths are in the range of 2.26–2.55 Å and 2.14–2.29 Å, respectively. And the Gd⋯Gd, Gd⋯Mn, and Mn⋯Mn separations (ranging from 3.85–3.95 Å, 3.53–3.67 Å, and 3.16–3.32 Å, respectively) are also comparable to those of other similar species.^37^ A space-filling diagram for 1 in Fig. 1 and S3 reveals that the torus has dimensions of about 2.6 nm on the long axis, about 2.3 nm on the short axis, and about 1.4 nm in thickness. The stacking structures for 1 are shown in Fig. S4.

For complex 4, its colorless product crystallizes in the tetragonal space group *P*4̄ (Table S1). The BVS calculation result and the colour of the compound revealed that the oxidation state for Mn centres is divalent. And the ring-like skeleton has crystallographic *C*_2v_ symmetry and is composed of sixteen Gd(iii) ions and sixteen Mn(ii) ions. To the best of our knowledge, 4 has the highest nuclearity in Mn(ii)–Ln clusters thus far.^31^ As presented in Fig. 1 and 2, the ring-like core of 4 can be viewed as being constructed from four kinds of [Mn_2_Gd_2_] sub-units (unit 1, unit 2, unit 3 and unit 4) (Fig. 2). Similar to the units in complex 1, the four [Mn_2_Gd_2_] units also closely resemble the [Mn_4_Ca] structure.^26^ All the deprotonated dpga^2−^ ligands adopt the 3.121 coordination modes to further connect adjacent building blocks. Besides, eight acetates also provide the key linkages. The coordination geometries and the SHAPE calculations result for Mn(ii) ions and Gd(iii) ions are shown in Table S6. The distances between neighboring metal ions (Gd⋯Gd = 3.86–3.91 Å, Gd⋯Mn = 3.53–3.62 Å, and Mn⋯Mn = 3.25–3.33 Å) are close to the ones in 1. And the Mn–O and Gd–O distances range from 2.11–2.28 Å and 2.27–2.50 Å, respectively, which are comparable to those in the reported complexes. The space-filling diagram for 2 reveals that the external and internal diameters of the cyclic molecule are *ca.* 3.2 nm and 0.7 nm, respectively, and thickness is *ca.* 1.4 nm (Fig. 1 and S5). The stacking structures for 4 along the *a* and *c* axes are shown in Fig. S6.

To assess the thermal stability of clusters 1 and 4, thermogravimetric analyses (TGA) were performed from 30 to 800 °C. As illustrated in Fig. S7, weight losses of 6.47% and 7.88% from room temperature to 170 °C are attributed to the removal of guest molecules (calcd 6.50% and 7.46%, respectively). Both compounds decompose rapidly above 190 °C. Under ultrasonic irradiation, crystals of 1 and 4 dissolved readily in dimethyl sulfoxide (DMSO), enabling electrospray ionization mass spectrometry (ESI-MS) to probe solution-phase integrity. High-quality ESI-MS data were successfully acquired for complex 1, but not for 4 which may be attributed to its higher molecular mass and poor ionization efficiency. The ESI-MS spectrum of 1 displays three dominant peaks at *m*/*z* 1102.83, 1174.75 and 1689.78 assigned to [Mn_12_Gd_12_(µ_3_-OH)_2_(µ_3_-OCH_3_)_22_(dpga)_12_(mmt)_2_(CH_3_COO)_4_(CH_3_OH)_3_(H_2_O)_2_]^6+^ (calcd 1101.08), [Mn_12_Gd_12_(µ_3_-OH)_2_(µ_3_-OCH_3_)_22_(dpga)_12_(mmt)_2_(CH_3_COO)_4_(CH_3_COOH)_3_ (CH_3_OH)_11_(H_2_O)_2_]^6+^(calcd 1173.83) and [Mn_12_Gd_12_(µ_3_-OH)_2_(µ_3_-OCH_3_)_22_(dpga)_12_(mmt)_2_(CH_3_COO)_6_(CH_3_OH)_4_(H_2_O)_2_]^4+^ (calcd 1689.16), respectively. Additionally, powder X-ray diffraction (PXRD) measurements were performed to confirm the phase purity of the as-synthesized samples of 1 and 4 (Fig. S9). Solution-phase stability was further assessed by UV-vis spectroscopy. As shown in Fig. S10, the UV-vis absorption spectra of 1 and 4 in H_2_O/DMSO solutions exhibit two characteristic bands at 253 and 294 nm, which can be assigned to ligand-to-metal charge-transfer transitions. Importantly, the absorption intensities remained essentially unchanged after five hours of visible-light irradiation, further corroborating their structural robustness in solution.

### Photocatalytic hydrogen production

**Fig. 3:**
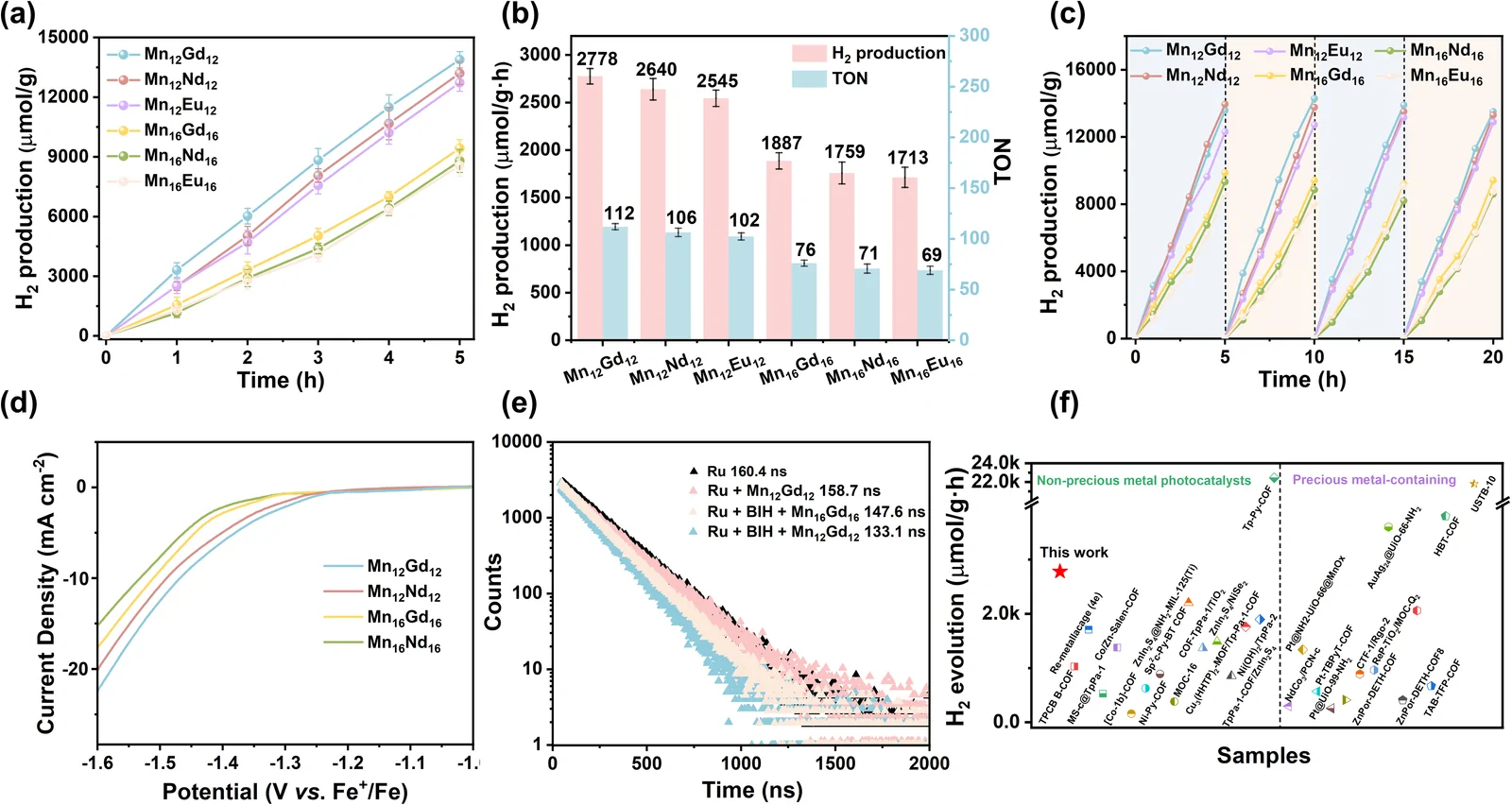
(a) Time-dependent H_2_ evolution of six clusters. (b) H_2_ evolution rate and TON of the six clusters. (c) Recycling experiments of the six clusters in four runs. (d) LSV curves of four clusters. (e) Time-resolved fluorescence decay spectra of [Ru(bpy)_3_]Cl_2_ (0.01 mM) in the presence of 0.2 mM BIH and 0.03 mM catalysts. (f) Comparison of H_2_ production performance between 1 and previously reported photocatalysts (including both precious metal-containing and precious metal-free systems).

Based on the structural features and solution stability of 1 and 4, their photocatalytic hydrogen production performance was evaluated. Hydrogen evolution experiments of 1–6 were conducted in a 20 mL mixture of H_2_O/DMSO (v/v = 19 : 1) under Xe-lamp irradiation, using 1,3-dimethyl-2-phenyl-2,3-dihydro-1*H*-benzimidazole (BIH) as the sacrificial electron donor and [Ru(bpy)_3_]Cl_2_·6H_2_O as the photosensitizer. As shown in Fig. 3a, hydrogen evolution gradually increased with irradiation time, yielding cumulative amounts of 13 883 and 9433 µmol g^−1^ after 5 h for 1 and 4, respectively. Notably, cluster 1 achieved the highest average hydrogen evolution rate of 2778 µmol g^−1^ (Fig. 3b), surpassing those of many reported Pt-free organic and inorganic photocatalysts (Fig. 3f and Table S3). Under 420 nm irradiation, the corresponding turnover number (TON) and apparent quantum yield (AQY) for cluster 1 were determined to be 112 and 0.474%, respectively. Furthermore, substitution of the lanthanide ion in 1 and 4 with neodymium (Nd) or europium (Eu) yielded analogues exhibiting hydrogen evolution activities comparable to those of the gadolinium-based parent complexes, indicating minimal influence of the lanthanide identity. The durability of the clusters during prolonged photocatalysis was evaluated by cyclic hydrogen evolution (Fig. 3c). Activity remained unchanged over four consecutive cycles, confirming excellent photostability of 1–6 under reaction conditions. Comparative experiments have shown that the cluster catalyst, the photosensitizer, the sacrificial agent, and light irradiation are all indispensable for the photocatalytic hydrogen production reaction (Fig. S11). UV-Vis spectroscopy further showed no change in the characteristic absorption peak intensity with extended reaction time (Fig. S12), indicating exceptional structural stability of 1–6. Furthermore, NMR spectroscopic analysis confirmed that the cluster structure was retained intact after catalysis (Fig. S13–S15).

To elucidate the catalytic mechanism of the aforementioned clusters, cyclic voltammetry (CV) measurements were conducted, as presented in Fig. S16 and S17. The CV profiles of 1 and 4 and their derivatives exhibit distinct reduction peaks near −1.2 V, accompanied by a shoulder at approximately −0.85 V. This potential region corresponds to the electrochemical reduction of protons in aqueous solution, suggesting that these clusters are capable of reducing protons in their reduced state.^38,39^ Further linear sweep voltammetry (LSV) analyses revealed that 1 delivers the lowest overpotential for hydrogen evolution, underscoring its superior proton–reduction activity relative to 4 and their derivatives (Fig. 3d).^40,41^ To probe the pathways of photoinduced electron transfer during photocatalysis, photoluminescence (PL) quenching and time-resolved PL decay measurements were performed on [Ru(bpy)_3_]^2+^ solutions in the presence of 1 (Fig. 3e). Stern–Volmer analysis showed nearly linear quenching behavior when BIH was employed as the quencher at varying concentrations. In contrast, no appreciable change in the fluorescence intensity of [Ru(bpy)_3_]^2+^ was observed upon addition of 1 (Fig. S18 and S19), indicating that 1 operates *via* a reductive quenching mechanism rather than an oxidative one.^42–44^ Moreover, comparative analysis of the PL lifetime decay kinetics demonstrated that, in [Ru(bpy)_3_]^2+^/BIH solutions, the introduction of 1 induces a more pronounced decrease in PL lifetime than does 4. This implies a higher electron transfer efficiency from photoexcited [Ru(bpy)_3_]^2+^ to 1 compared to 4.^45^ Collectively, these findings establish that 1 possesses markedly superior overall electron transport properties as a photocatalyst relative to 4, thereby enabling more efficient photogenerated charge transfer within the photocatalytic H_2_-evolution system.

The photocatalytic hydrogen evolution performance of the Mn–Ln clusters is closely related to their nuclearity, lanthanide identity, and electronic properties. Clusters with higher nuclearity provide more metal active sites and extended Mn–O–Ln connectivity, which can facilitate charge migration and improve the utilization of photogenerated charge carriers. Meanwhile, different lanthanide ions exhibit distinct ionic radii and 4f electronic configurations, resulting in variations in the electronic structures and metal–metal interactions within the clusters. These structural and electronic differences influence charge separation and transfer processes, ultimately affecting photocatalytic hydrogen evolution activity.^4,11–13^

**Fig. 4:**
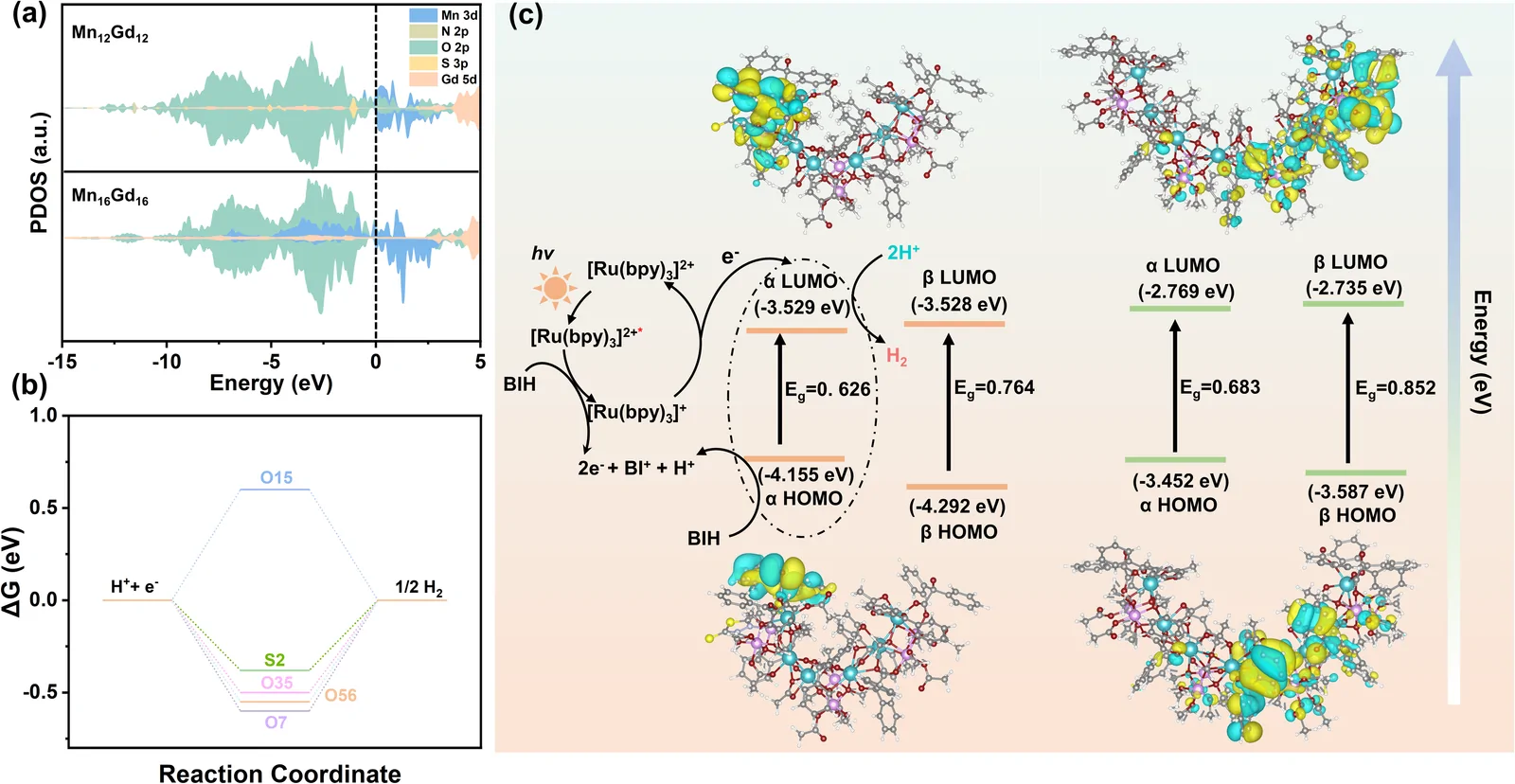
(a) Projected density of states (PDOS) of clusters 1 and 4 (the Fermi level is defined at 0 eV). (b) Gibbs free energy profiles of the HER for 1. (c) DFT-calculated HOMO and LUMO energy levels and the proposed photocatalytic mechanism.

To gain deeper insight into the photocatalytic hydrogen evolution mechanism of 1 and 4, density functional theory (DFT) calculations were carried out. First, the projected density of states (PDOS) for both clusters was computed and analyzed. As illustrated in Fig. 4a, the PDOS of 1 exhibits pronounced orbital overlap between the Mn 3d and O 2p orbitals near the Fermi energy level (EF), signifying strong electronic coupling between the central Mn ion and the oxygen atoms of the ligands.^46^ In contrast, the Mn 3d and O 2p orbital contributions at EF in 4 are markedly diminished. This finding aligns with the superior photocatalytic hydrogen evolution performance observed for 1. Additionally, DFT calculations were employed to identify the catalytically active sites involved in the proton reduction reaction. As shown in Fig. S20, various candidate structural sites across 1 and 4 were systematically evaluated. As depicted in Fig. 4b, the atom exhibiting the lowest Gibbs free energy change (Δ*G*) for hydrogen adsorption in 1 is the sulfur atom (S2) on the mmt^−^ ligand (Δ*G* = −0.38 eV), whereas in 4 it is the oxygen atom (O57) (Δ*G* = −0.40 eV). These values indicate that S2 and O57 serve as thermodynamically favorable sites for proton adsorption and subsequent reduction to H_2_.^47^ Notably, the S2 site on the mmt^−^ ligand in 1 functions as the primary active center for proton reduction during photocatalytic hydrogen production-exhibiting higher intrinsic activity than the O57 site in 4 (Fig. S21). Furthermore, analysis of the electronic density distributions reveals that the highest occupied molecular orbital (HOMO) of both clusters is predominantly localized on ligand moieties distal to the Mn(ii) ions, whereas the lowest unoccupied molecular orbital (LUMO) is primarily distributed over ligand regions proximal to the Mn(ii) centers. This spatial separation between the HOMO and LUMO promotes efficient intramolecular charge transfer, thereby endowing both clusters with high charge separation efficiency. Owing to the spin polarization effect of Mn(ii) ions, two distinct sets of orbital energy levels-denoted as α and β-were computed (Fig. 4c). The HOMO–LUMO energy gap of 1 is markedly narrower than that of 4, indicating that 1 exhibits enhanced facilitation of electron transport.^9,48^

Based on the above conclusions, a mechanistic pathway for photocatalytic hydrogen evolution over 1 is proposed (Fig. 4c). Upon photoexcitation, the excited-state [Ru(bpy)_3_]^2+^ photosensitizer (PS) generates electron–hole pairs. The photogenerated electrons are subsequently injected into the LUMO of cluster 1, which is mainly localized around the Mn centers, indicating that the Mn sites serve as the primary electron-accepting centers during the photocatalytic process. The accumulated electrons at the Mn centers can further migrate through the Mn–O–Ln framework, promoting intracluster charge transfer and regulating the electronic environment of the ligand framework. According to DFT calculations, H adsorption preferentially occurs at the oxygen atoms of the ligand, suggesting that these O sites provide favorable adsorption environments for proton reduction and H_2_ evolution. Meanwhile, the sulfur- and oxygen-containing ligand framework facilitates charge delocalization and stabilizes the reaction intermediates during catalysis. The photogenerated holes are consumed by BIH *via* an oxidative quenching pathway. Therefore, the efficient photocatalytic hydrogen evolution activity of cluster 1 can be attributed to the cooperative contribution of Mn-based electron-transfer centers and O-containing ligand sites, which together promote charge separation, electron migration, and proton reduction.

## Conclusions

In conclusion, two families of nanostructured CCCs {Mn_12_Ln_12_} and {Mn_16_Ln_16_} have been isolated. Each cluster is assembled from repeating {Mn_2_Ln_2_O_4_} cubane-like metal-oxo units, which contribute to the formation of the overall cluster architecture. To our knowledge, {Mn_16_Ln_16_} stands as the highest-nuclearity cluster among the reported Mn(ii)–Ln clusters. Both families exhibit good solubility in organic/aqueous mixed solvents and efficient photocatalytic hydrogen production. Notably, cluster {Mn_16_Gd_16_} achieves a hydrogen evolution rate exceeding those of numerous reported photocatalysts, including both precious-metal-containing and precious-metal-free systems. This work provides a promising foundation for rationally designing high-performance Mn-based molecular photocatalysts for H_2_ production.

## Author contributions

Y.-Z. Z. designed and supervised the project. K.-P. B. and Y. W. were responsible for the synthesis and basic characterization experiments. K.-P. B. and W.-P. C. carried out structural analysis of the six complexes and performed the investigations in photocatalytic hydrogen production. W.-P. C. provided advice and support for experiments. Q.-C. L. and Y.-Q. Z. carried out part of the calculations and analysis. X.-Q. H. provided the financial and technical support. K.-P. B. wrote the original draft. All authors contributed in discussing the results and commented on the manuscript.

## Conflicts of interest

There are no conflicts to declare.

## Supplementary Material

SC-OLF-D6SC06444B-s001

SC-OLF-D6SC06444B-s002

## Data Availability

The data supporting the findings of this study are available from the corresponding author upon reasonable request. CCDC 2269142 and 2269144 contain the supplementary crystallographic data for this paper.^49*a*,*b*^ Supplementary information (SI): experimental procedures, structure characterization data, and theoretical computational methodologies. See DOI: https://doi.org/10.1039/d6sc06444b.
