# Supplementary material for: Assembling giant heterometallic cyclic coordination clusters {MnII2LnIII2}n (n = 6 and 8) for efficient photocatalytic hydrogen production
Source: Chem Sci. 2026 Sep 15. Online ahead of print. doi: 10.1039/d6sc06444b (PMC13592353; doi:10.1039/d6sc06444b)
Supplement: Supplementary file 1 [file SC-OLF-D6SC06444B-s001.pdf]

Supplementary Information (SI) for Chemical Science.
This journal is © The Royal Society of Chemistry 2026

Supporting Information

Assembling Giant Heterometallic Cyclic Coordination Clusters $\{Mn^{II}_2Gd^{III}_2\}_n$ (n = 6 and 8) for Efficient Photocatalytic Hydrogen Production

Kai-Peng Bai,[#a] Yu Wang, [#a] Wei-Peng Chen, [*b] Yuan-Qi Zhai,[a] Qian-Cheng Luo,[a] Xiang-Quan Hu,[a] and Yan-Zhen Zheng[*a]

[a] Dr. K.-P. Bai, Y Wang, Dr. Y.-Q. Zhai, Dr. Q.-C. Luo, Prof. X.-Q Hu, Prof. Y.-Z. Zheng
Frontier Institute of Science and Technology, Interdisciplinary Research Center of Frontier science and technology, State Key Laboratory of Electrical Insulation and Power Equipment, Xi'an Key Laboratory of Electronic Devices and Materials Chemistry, School of Future Technology, and School of Chemistry, Xi'an Jiaotong University, Xi'an 710054, China.

[b] Prof. W.-P. Chen
School of Materials Science and Engineering, Shaanxi University of Technology, Hanzhong 723001, China

#. Contributed equally to this work.

Corresponding Author:
*Email: zheng.yanzhen@xjtu.edu.cn (Y.-Z. Zheng); sxchenweipeng@163.com (W.-P Chen)

Table of Contents

Experiment Section 4

Scheme S1. Schematic illustration of the solvothermal synthesis of [$Mn_{12}Gd_{12}$] and [$Mn_{16}Gd_{16}$]. 5

Supporting Tables and Figures 10

Table S1. Crystal Data and Structure Refinement for complexes **1** and **4**. 10

Table S2. Bond Valence Sum Calculation for the manganese centers in complexes **1** and **4.** 11

Figure S1. Infrared spectra for complexes **1**(a) and **4**(b). 12

Figure S2. The coordination modes of $dpga^{2-}$, mmt^{-} and CH_3COO^{-} ligands. 13

Figure S3. Ball-and-stick views and space filling diagrams for complex **1**.. 14

Figure S4. The stacking structures for **1** along the *a*, *b* and *c* axis, respectively. 15

Figure S5. Ball-and-stick views and space filling diagrams for complex **4.** 16

Figure S6. The stacking structures for **4** along the *a* and *c* axis, respectively. 17

Figure S7. Thermogravimetric analysis for **1** and **4**. 18

Figure S8. Positive-mode ESI-MS spectrum of **1** in dimethyl sulfoxide solution. 19

Figure S9. The PXRD patterns of **1-6**. 20

Figure S10. UV-vis spectra of 0.03 mM different catalysis in H_2O/DMSO (20 mL, v/v = 19:1) after the irradiation of 0-5 h 21

Figure S11. Photocatalytic H_2 production activity of **1** under the altered conditions. 22

Figure S12. UV-vis spectra of 0.03 mM different catalysis, 20 mg photosensitizer [Ru(bpy)$_3$]Cl$_2$·6H$_2$O, 40 mg BIH in H_2O/DMSO (20 mL, v/v = 19:1) after the irradiation of 0-5 h.. 23

Figure S13. 1H NMR spectra of [Ru(bpy)$_3$]Cl$_2$·6H$_2$O (a), BIH (b) and ligand H_2dpga (c). 24

Figure S14. 1H NMR spectra of **1**. 25

Figure S15. 1H NMR spectra of **4**.. 26

Figure S16. Cyclic voltammogram of the four clusters. 27

Figure S17. Potentials of Ag/AgCl are referenced to the ferrocene/ferrocenium couple. 27

Figure S18. (a) The steady-state fluorescent spectra of [Ru(bpy)$_3$]Cl$_2$ (0.01 mM) upon the addition of increasing amounts of BIH (λex = 415 nm). (b) Stern-Volmer plot of the emission quenching of [Ru(bpy)$_3$]Cl$_2$ solution by BIH. 28

Figure S19. (a) The steady-state fluorescent spectra of [Ru(bpy)$_3$]Cl$_2$ (0.01 mM) upon the addition of increasing amounts of $Mn_{12}Gd_{12}$ (λex = 415 nm). (b) Stern-Volmer plot of the emission quenching of [Ru(bpy)$_3$]Cl$_2$ solution by $Mn_{12}Gd_{12}$. 29

Figure S20. The different adsorption sites in cluster **1** 30

Figure S21. Local density maps of **1** (a) and **4** (b), and Gibbs free energies at different adsorption sites. 31

Table S3. Comparison of the photocatalytic hydrogen production performance of several organic or inorganic catalysts systems. 32

Table S4. The CShM calculations of hexa-coordinate Mn(II) ions in **1**. 33

Table S5. The CShM (Continuous Shape Measures) calculations of octa-coordinate Gd(III) ions in **1**. 34

Table S6. The CShM calculations of hexa-coordinate Mn(II) ions in **4**. 35

Table S7. The CShM calculations of octa-coordinate Gd(III) ions in **4**. 36

Table S8. Selected bond distances (Å) for **1**. 37

Table S9. Selected bond distances (Å) for **4**. 38

References 39

Experiment Section

1. Materials and Measurements

All reagents were used as received from commercial suppliers without further purification. Elemental analyses (EA) for carbon, hydrogen, and nitrogen were performed on a EUROVECTOR EA3000 elemental analyzer (Euro Vector S.p.A.). Infrared (IR) spectra (4000–500 cm^{-1}) were recorded on a Thermo Scientific Nicolet 6700 FT-IR spectrophotometer. Thermogravimetric analysis (TGA) was carried out on a METTLER TOLEDO TGA-2 thermogravimetric analyzer under a nitrogen atmosphere, with heating from 30 to 800 ^{o}C. Powder X-ray diffraction (PXRD) patterns were collected at room temperature using a Rigaku SmartLab diffractometer equipped with Cu Kα radiation (45 kV, 40 mA) and a diffracted-beam graphite monochromator. UV–Vis diffuse reflectance spectra were acquired on a PerkinElmer Lambda 950 spectrophotometer. ^{1}H NMR spectra were recorded on a Bruker AVANCE NEO 400 spectrometer. Photoluminescence (PL) spectra were measured using a Hitachi F-4600 fluorescence spectrophotometer. Time-resolved PL decay profiles were obtained with an Edinburgh Instruments FLS980 fluorescence lifetime spectrophotometer. Gas-phase reaction products were identified and quantified by gas chromatography (Agilent Technologies 7890B). High-resolution electrospray ionization mass spectrometry (HR-ESI-MS) was performed on a Thermo Scientific Exactive mass spectrometer using H_2O/DMSO solutions.

2. Syntheses

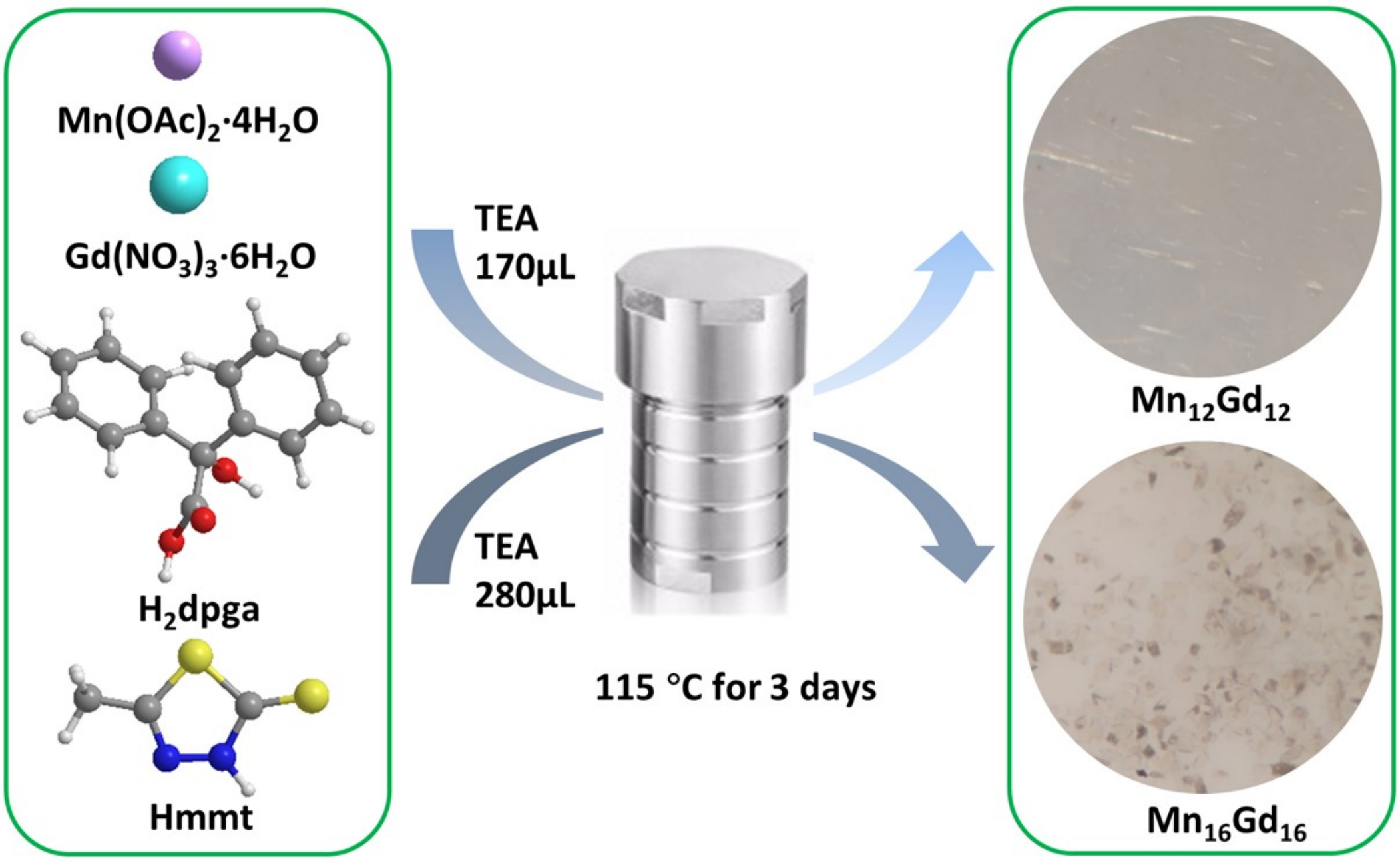

Scheme S1. Schematic illustration of the solvothermal synthesis of [$Mn_{12}Gd_{12}$] and [$Mn_{16}Gd_{16}$] via hydrolysis of $Mn(OAc)_2 \cdot 4H_2O$ and $Gd(NO_3)_3 \cdot 6H_2O$ in a methanol–acetonitrile mixture, using triethylamine as a base and diphenylglycolic acid (H_2dpga) and 2-mercapto-5-methyl-1,3,4-thiadiazole (Hmmt) as co-ligands.

Synthesis of [$Mn^{II}_{12}Gd^{III}_{12}(\mu_3\text{-}OH)_2(\mu_3\text{-}OCH_3)_{22}$(dpga)$_{12}(mmt)_2(CH_3COO)_{10}(CH_3COOH)_6(CH_3OH)_{12}(H_2O)_2$]·$6CH_3OH·5CH_3CN·7H_2O$ (1)

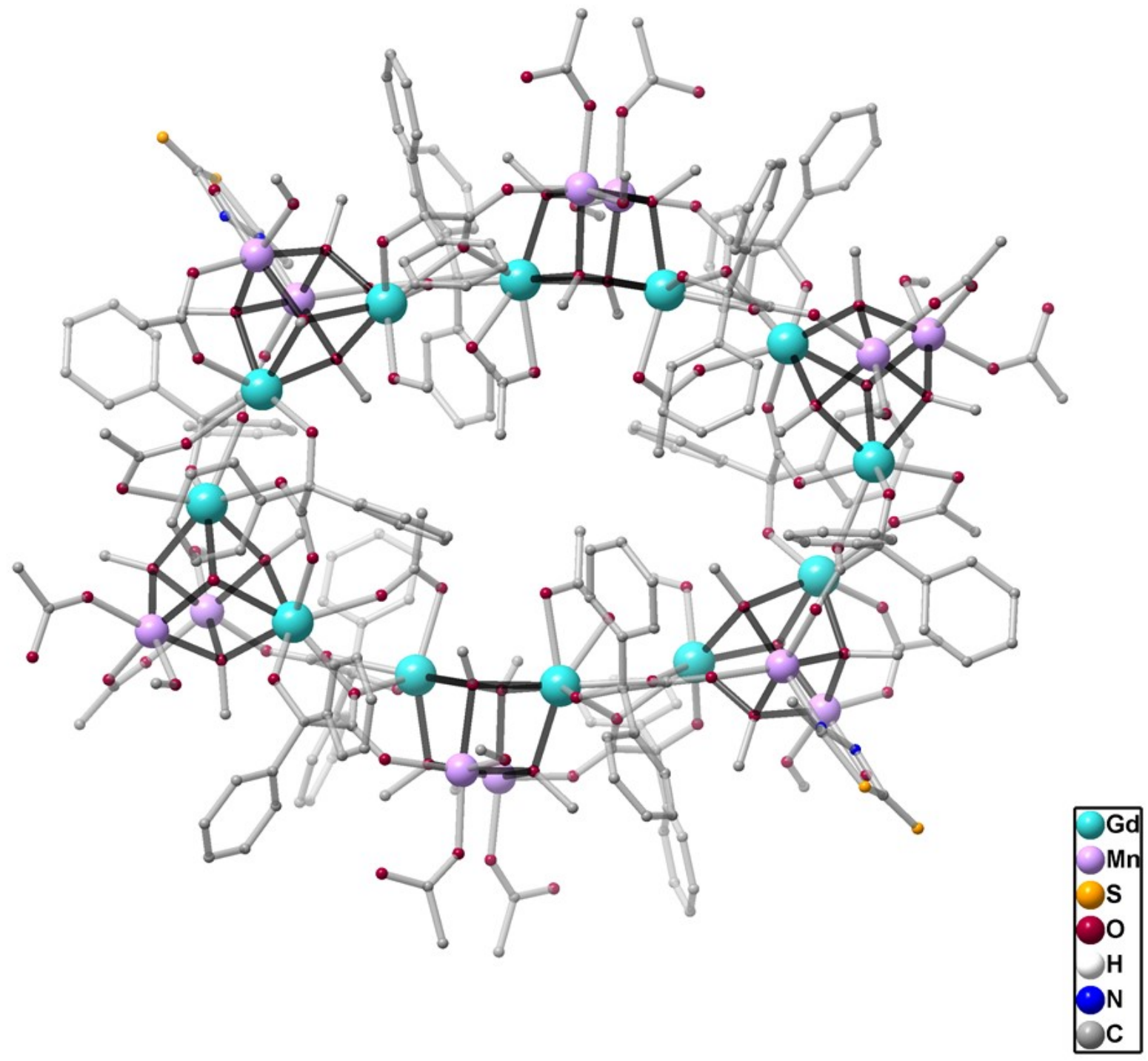

A mixture of 2-Mercapto-5-methyl-1,3,4-thiadiazole (Hmmt, 0.07 g, 0.5 mmol), diphenylglycolic acid (H_2dpga, 0.11 g, 0.5 mmol), $Mn(CH_3COO)_2·4H_2O$ (0.16 g, 0.65mmol), $Gd(NO_3)_3·6H_2O$ (0.225 g, 0.5 mmol), KBr (0.05 g, 0.42 mmol) and triethylamine (1.2 mmol, 0.17 ml) were dissolved in the mixed solvent of CH_3OH/CH_3CN (v/v = 4 ml: 4 ml). The mixed solution was then sealed in a 12 mL Teflon-lined stainless-steel vessel and heated at 115 °C for 3 days. At a rate of 5 °C/h, the system was allowed to cool to room temperature. Colorless needle-like crystals of **1** were collected with the yield of 41% (based on H_2mmt), washed with acetonitrile, and dried in air at room temperature. Elemental analysis calcd (%) for $C_{250}H_{337}Gd_{12}Mn_{12}N_9O_{119}S_4$: C 37.81, H 4.35, N 1.55. Found: C 37.45, H 4.34, N 1.62. IR (KBr, cm^{-1}): 3346 (w), 3057 (w), 2930 (w), 2820 (w), 2397 (m), 1764 (m), 1651 (w), 1570 (s), 1382 (s), 1210(w), 1167 (w), 1098 (m), 1065 (w), 1024 (s), 944 (w), 914 (w), 825 (s), 810 (m), 761 (w), 733 (w), 701 (s), 680 (m), 647 (w), 605 (m).

Synthesis of $[Mn^{II}_{16}Gd^{III}_{16}(\mu_3\text{-}OCH_3)_{32}(dpga)_{16}(CH_3COO)_{16}(CH_3COOH)_8(CH_3OH)_{20}]\cdot 14H_2O\cdot 11CH_3CN\cdot 5CH_3OH$ (4)

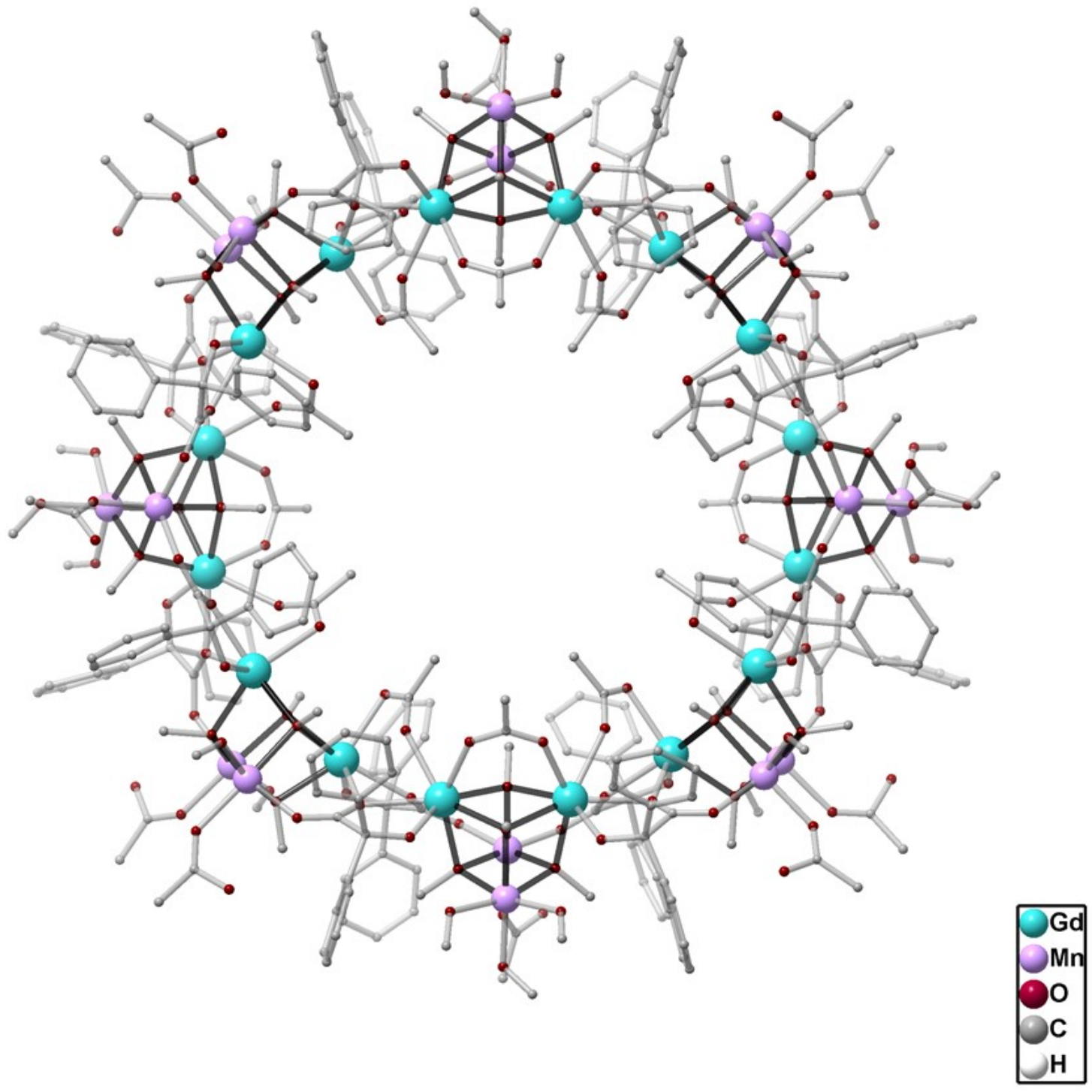

The complex **4** can be obtained by the same method for **1**, except that increase the amount of triethylamine to 2 mmol. Colorless block crystals were collected with the yield of 43% (based on Gd^{3+}). Elemental analysis calcd (%) for $C_{351}H_{497}Gd_{16}Mn_{16}O_{167}N_{11}$: C 38.54, H 4.58, N 1.41. Found: C 38.13, H 4.47, N 1.52. IR (KBr, cm^{-1}): 3604 (w), 3353 (w), 3062 (w), 2935 (s), 2826 (s), 1625 (s), 1490 (m), 1446 (m), 1382 (s), 1211 (w), 1171 (w), 1096 (w), 1070 (w), 1042 (s), 949 (w), 914 (w), 832 (w), 792 (m), 757 (w), 736 (w), 698 (s), 671 (w), 624 (s), 572 (m), 536 (w).

3. X-ray crystallography

Single-crystal X-ray diffraction data of **1** and **4** were collected at the Bruker APEX II diffractometer at 150 K. The structures were solved by direct methods and all non-H atoms were subjected to anisotropic refinement by full-matrix least-squares refinement on F^2 using Olex2 program. The electron count and void volume obtained from the SQUEEZE calculation. For compound **1**, the solvent mask analysis revealed a solvent-accessible void volume of 1584 Å^3 containing 384 electrons per unit cell. These values are consistent with the presence of 18 CH_3CN molecules per asymmetric unit, corresponding to 396 electrons per unit cell. For compound 4, the calculation revealed one solvent-accessible void per unit cell with a volume of 11235 Å^3 containing 4702 electrons. This electron count is consistent with the presence of approximately 60 CH_3CN and 50 CH_3OH molecules per asymmetric unit. The refinement parameters and crystallographic data for complexes **1** and **4** are shown in Table S1. Selected bond lengths are given in Table S10-S12. CCDC 2269142 and 2269144 contain the supplementary crystallographic data. This data can be obtained free of charge via www.ccdc.cam.ac.uk/data_request/cif.

4. Photochemical Experiments

The photocatalytic activities of all samples were carried out in a 250 mL quartz reactor with a top irradiation window. The reaction temperature was controlled at ambient temperature (25°C) by the circulation water system. The light source was a 300 W Xe lamp covered with a cut-off filter with a wavelength range of 420-800 nm. Screening for the best reaction conditions. The 0.03 mM catalyst (5 mg), 20 mg photosensitizer $[Ru(bpy)_3]Cl_2{\cdot}6H_2O$, along with 40 mg of sacrificial electron donor 1,3-dimethyl-2-phenyl-2,3-dihydro-1H-benzo[d]-imidazole (BIH), were sealed in 20 mL H_2O/DMSO ((*v*/*v* = 19:1). Before irradiation, Ar was passed through the solution to degas it. The generated gas samples were analyzed using an Agilent Technologies-7890B gas chromatography.

5. Electrochemical studies

The electrochemical behavior of **1** and **4** was investigated by using a three-electrode system. Cyclic voltammetry was performed with an electrochemical workstation CHI660E in H_2O/DMSO mixture (19:1 = v/v) containing 0.20 M Na_2SO_4 as an electrolyte at room temperature. Glassy Carbon (3 mm diameter) was used as working electrode, a platinum wire as auxiliary electrode, and an Ag/AgCl electrode as the reference electrode. The electrolyte solution was saturated with Ar by purging with Ar for 30 min prior to each experiment. Before the experiment, under

identical conditions, ferrocene is added to the solution, and its redox peak is measured to determine the half-cell potential of Fc/Fc^{+}. All measured potentials are then converted to values relative to Fc/Fc^{+}, by subtracting the $E_{1/2}$ of ferrocene.

6. Determination of the quantum yield

The apparent quantum Yield (AQY) for hydrogen evolution was measured under the illumination of a 300 W Xe lamp with bandpass filters for 1 h. The AQY were calculated using the following equation:

AQY = [2 × (number of the produced molecule) / (number of photons)] ×100%

$$AQY = \frac{(v \times N_A \times K) \times (h \times c)}{I \times A \times \lambda}$$

v is the reaction rate of product H_2; N_A is Avogadro's constant (6.02×10^{23} mol^{-1}); K is the number of electrons transferred by the reaction (2); h is the Planck's constant (6.63×10^{-34} m^2 kg s^{-1}); c is the speed of light (3×10^{8} $m\cdot s^{-1}$); I is the irradiation intensity (W cm^{-2}); A is the irradiation area (m^2); λ is the wavelength of the light source (420 nm).

7. Computational Details

First-principles calculations were performed within the framework of density functional theory (DFT) as implemented in the Vienna Ab initio Simulation Package (VASP)[1]. The exchange–correlation interactions were treated using the generalized gradient approximation (GGA) with the Perdew–Burke–Ernzerhof (PBE) functional[2]. Spin polarization was considered in all calculations to account for possible magnetic effects. Two cluster models were investigated: cluster 1 fragment (391 atoms) and cluster 4 fragment (501 atoms). The electronic wavefunctions were expanded in a plane-wave basis set with a kinetic energy cutoff of 520 eV, ensuring a balanced accuracy and computational efficiency. Geometry optimizations were carried out until the residual force on each atom was below 0.03 eV $Å^{-1}$, while the electronic self-consistent field (SCF) convergence criterion was set to 1×10^{-5} eV[3]. Brillouin-zone integrations were performed using a Γ-centered Monkhorst–Pack k-point mesh[4], suitable for these large cluster systems. To account for long-range van der Waals interactions, the DFT-D3 dispersion correction scheme was employed[5]. All calculations were conducted with careful convergence tests to ensure the reliability of the obtained structures and electronic properties.

Supporting Tables and Figures

Table S1. Crystal Data and Structure Refinement for complexes **1** and **4**.

**Complex**	$Mn_{12}Gd_{12}$ (**1**)	$Mn_{16}Gd_{16}$ (**4**)
**Formula**	$C_{256}H_{351}Gd_{12}Mn_{12}N_9O_{119}S_4$	$C_{351}H_{497}Gd_{16}Mn_{16}O_{167}N_{11}$
**Formula weight**	8133.02	10937.66
**Space group**	$P\bar{1}$	$P\bar{4}$
a **(Å)**	14.8617(7)	33.0415(6)
b **(Å)**	25.0753(9)	33.0415(6)
c **(Å)**	25.3487(1)	25.1978(7)
α **(°)**	113.493(1)	90
β **(°)**	102.763(2)	90
γ **(°)**	96.405(2)	90
**V(Å^3)**	8236.3(6)	27509.5(13)
Z	1	2
Dc **/g cm^{-3}**	1.560	1.213
μ **/ mm^{-1}**	2.921	2.311
F **(000)**	3810.0	9880.0
R **(int)**	0.0765	0.0978
GOOF	1.042	1.022
**Final *R* indices [*I*>2sigma(I)]**	R_1 = 0.0359, wR_2 = 0.0965	R_1 = 0.0581, wR_2 = 0.1319

$R_1 = \Sigma||F_0|-|F_c||/\Sigma|F_0$, $wR_2 = \{\Sigma[w(F_0^2-F_c^2)^2]/\Sigma[w(F_0^2)^2]\}^{1/2}$

Table S2. Bond Valence Sum Calculation for the manganese centers in complexes **1** and **4**.

**Compounds**	**Manganese centers**	**BVs assuming (Mn^{II})**	**BVs assuming (Mn^{III})**	**Designation**
{$Mn_{12}Gd_{12}$} (**1**)	Mn1	2.032	1.873	2
	Mn2	2.026	1.868	2
	Mn3	1.972	1.818	2
	Mn4	2.045	1.887	2
	Mn5	1.959	1.808	2
	Mn6	2.062	1.903	2
{$Mn_{16}Gd_{16}$} (**4**)	Mn1	2.077	1.913	2
	Mn2	2.160	1.992	2
	Mn3	2.038	1.879	2
	Mn4	2.002	1.846	2
	Mn5	1.981	1.825	2
	Mn6	2.001	1.846	2
	Mn7	1.918	1.769	2
	Mn8	1.970	1.817	2

The bond-valence-sum (BVS) analysis was performed to verify the oxidation state of the manganese centers in compounds **1** and **4**. The calculation formula is $s = \exp[(r_0 - r)/B]$, s is the valence of the individual bond, r is the observed bond length, r_0 is a constant dependent upon the bonded elements (Mn^{2+}-O, r_0 = 1.790; Mn^{2+}-N, r_0 = 1.862; Mn^{3+}-O, r_0 = 1.760; Mn^{3+}-N, r_0 = 1.832) and b is a constant of 0.37 (*Inorg. Chem*. **1991**, *31*, 1585). As shown in above Table, all of the manganese ions are +2 valent by BVS calculations. Moreover, the colours of crystals are also particularly characteristic of oxidation state for manganese centers because the Mn(II) ions prefer to form colorless crystals.

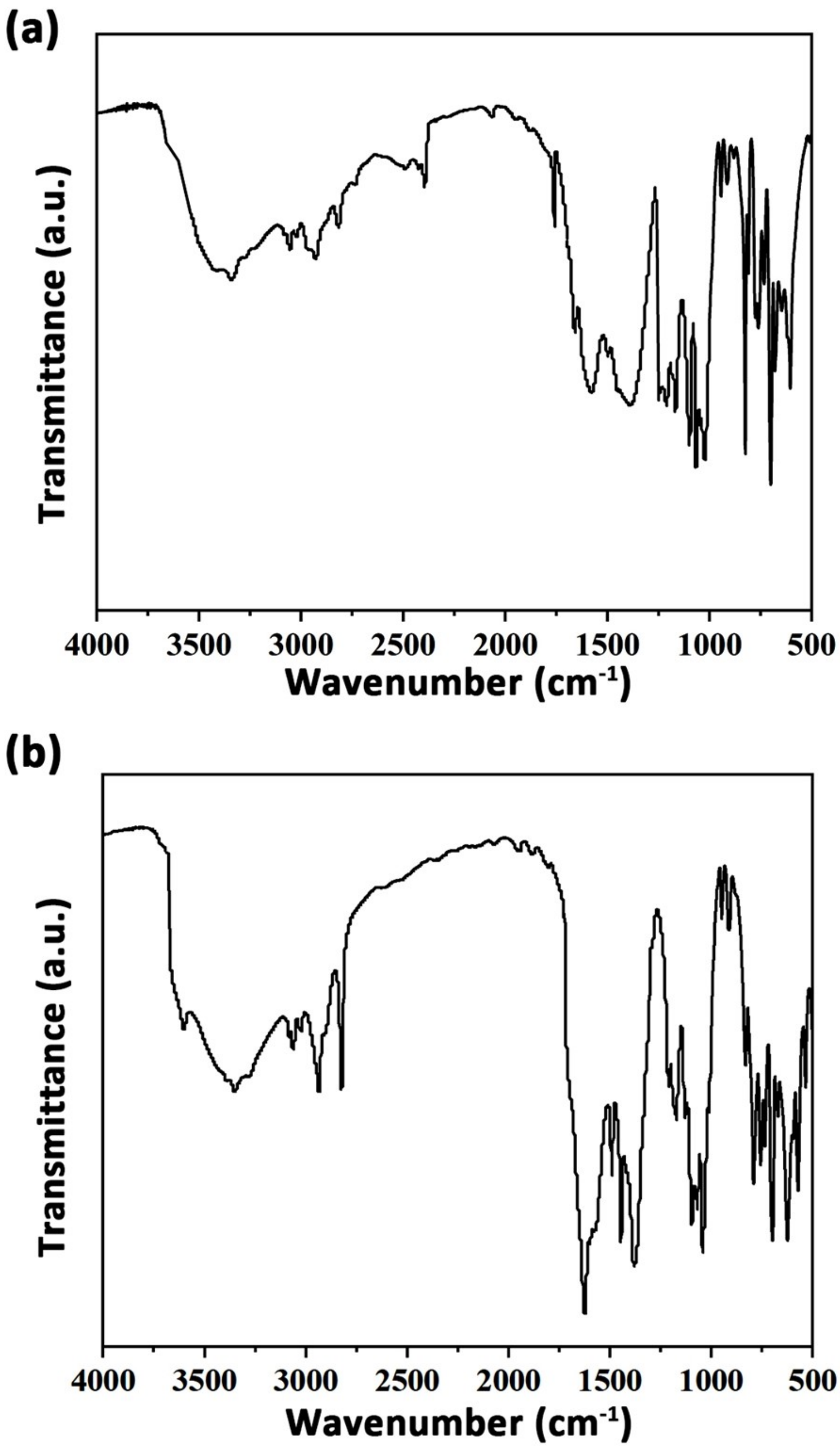

Figure S1. Infrared spectra for complexes **1**(a) and **4**(b).

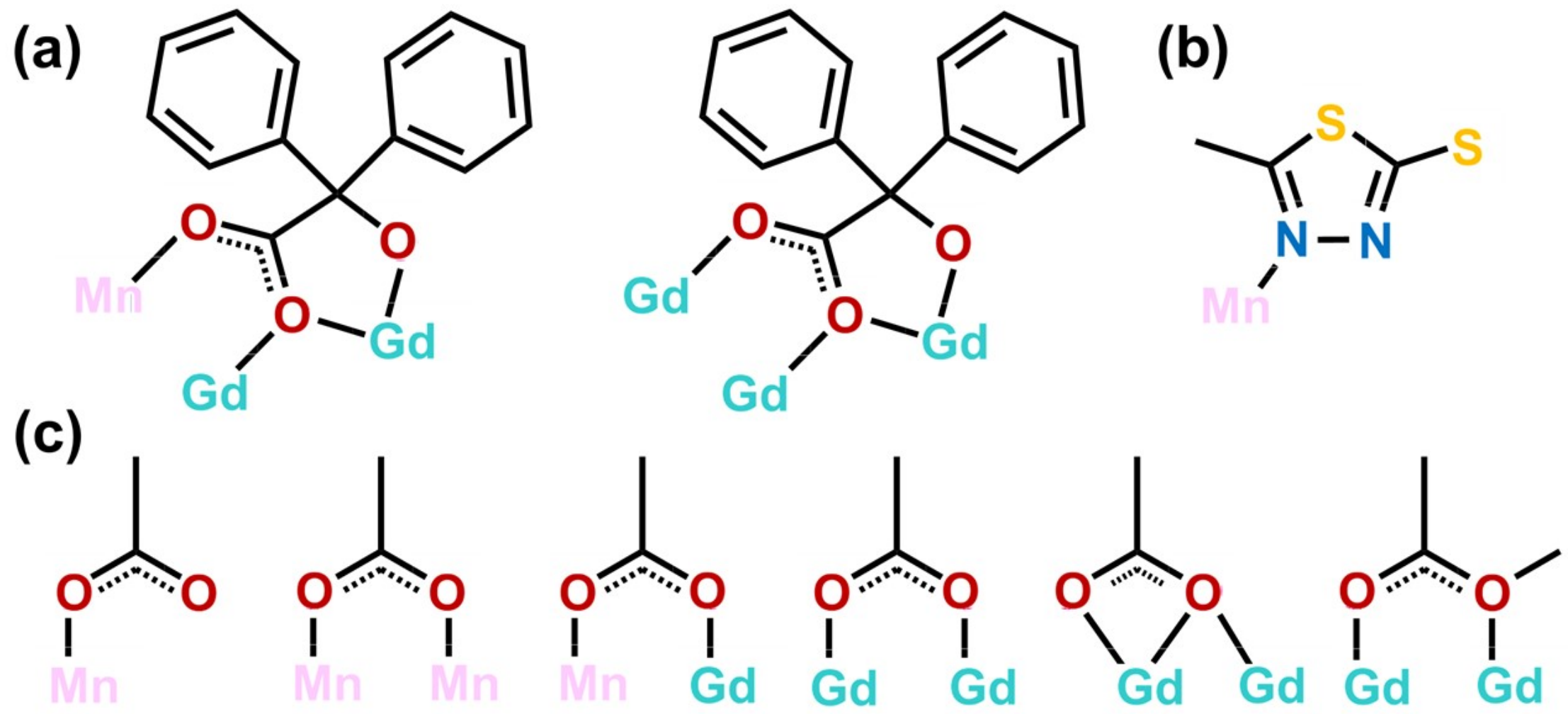

Figure S2. The coordination modes of dpga^{2-} (a), mmt^{-} (b) and CH_3COO^{-} (c) ligands in clusters **1** and **4**.

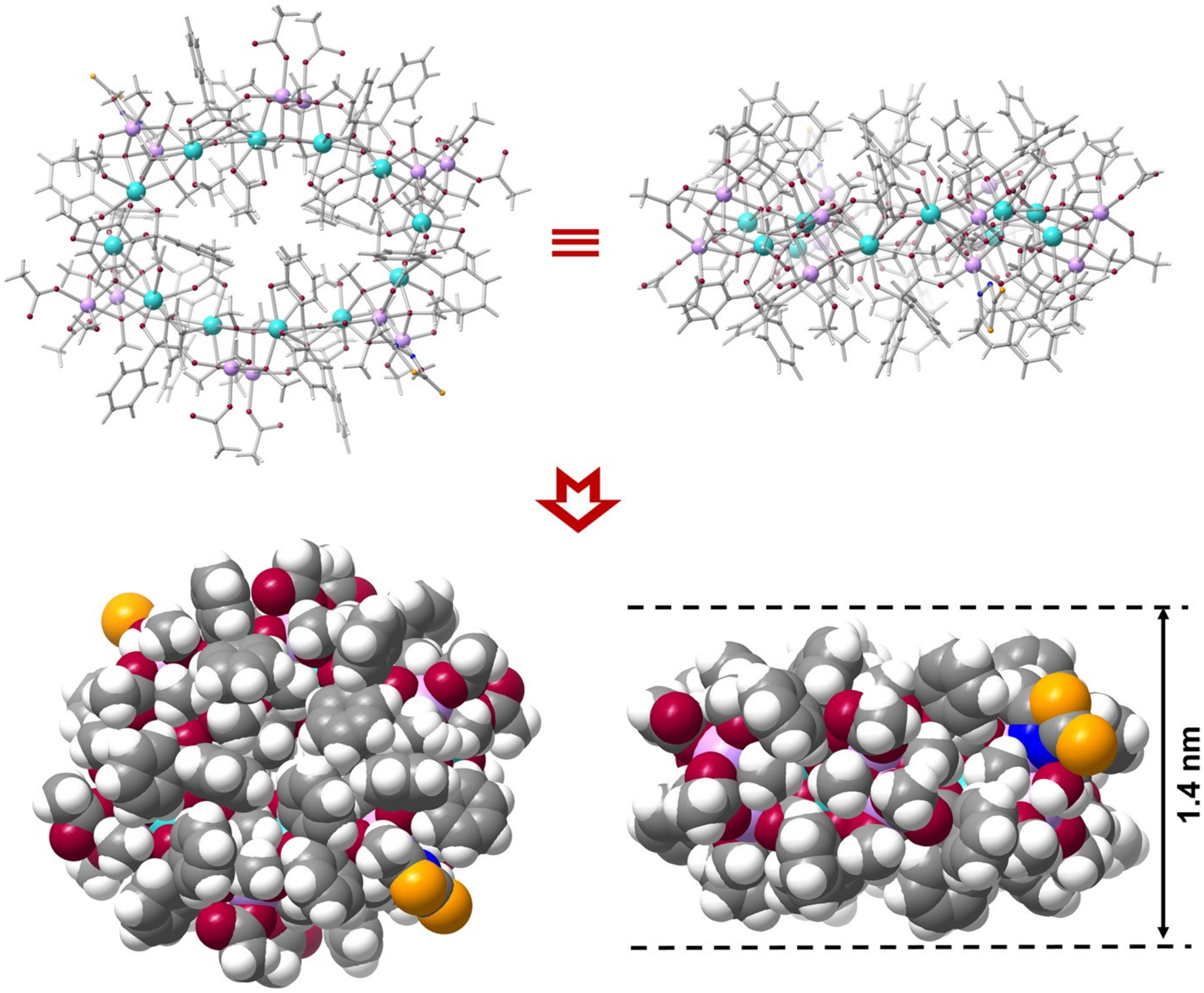

Figure S3. Ball-and-stick views and space filling diagrams for complex **1**. Color codes: Gd, cyan; Mn(II), pink; S, yellow; N, blue; O, red; C, grey; H, white.

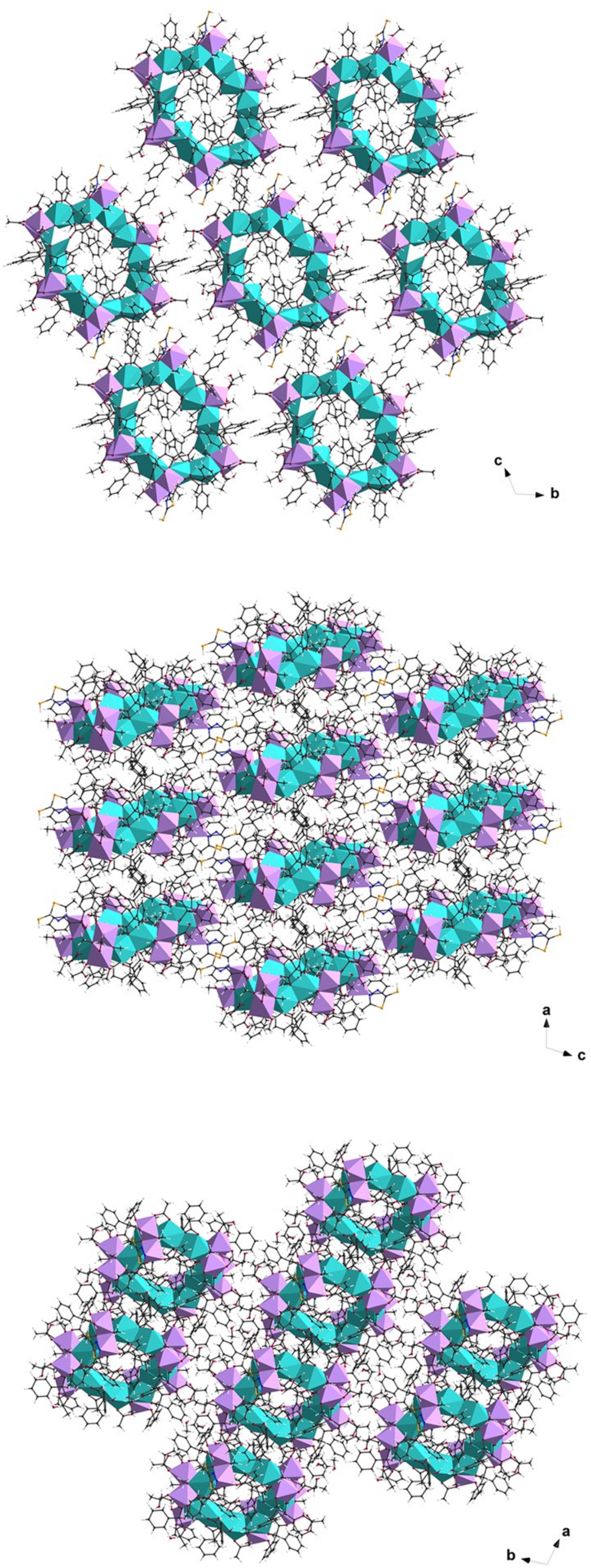

Figure S4. The stacking structures for **1** along the *a*, *b* and *c* axis, respectively. Color codes: Gd, cyan; Mn(II), pink; S, yellow; N, blue; O, red; C, grey; H, white.

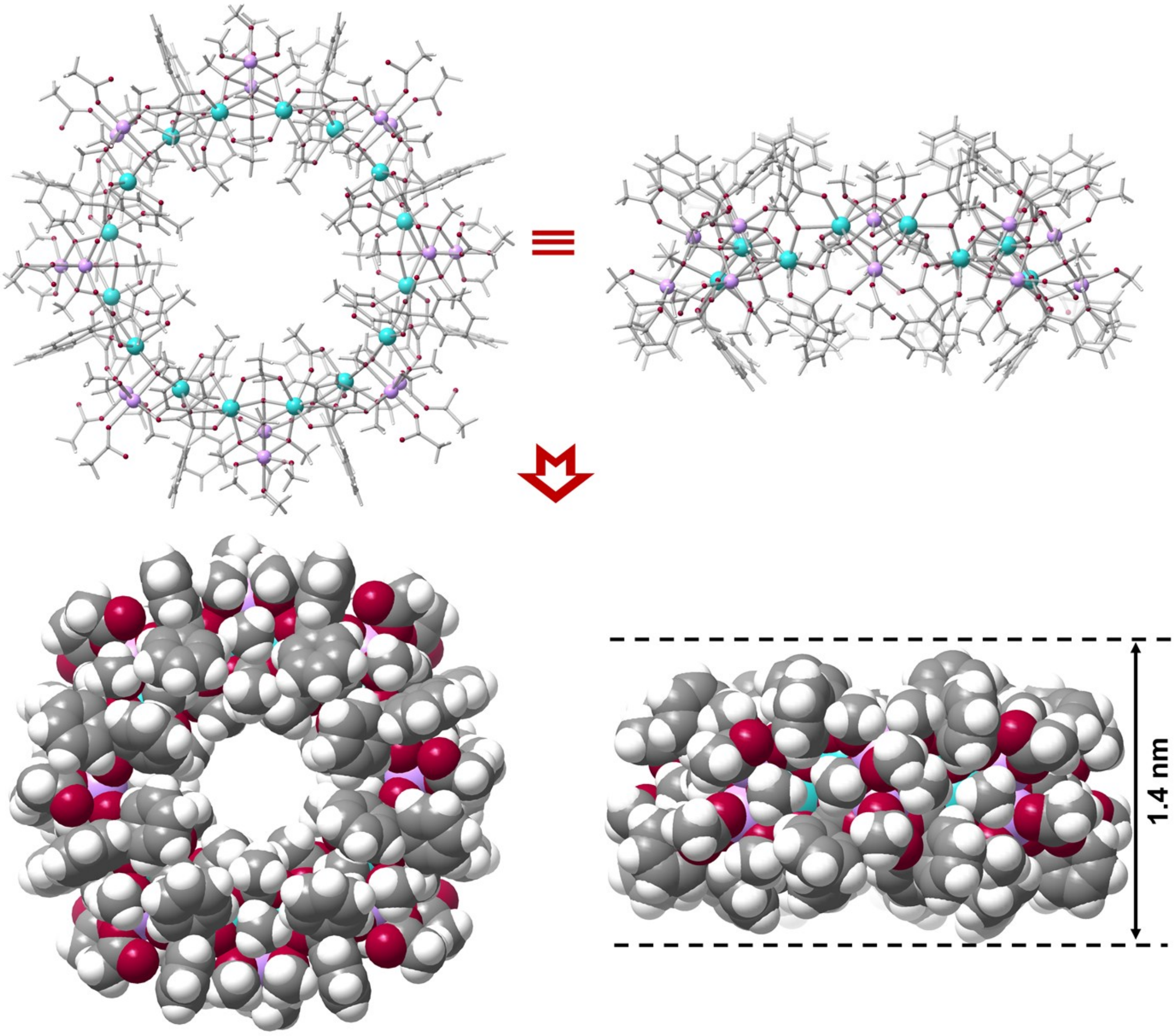

Figure S5. Ball-and-stick views and space filling diagrams for complex **4**. Color codes: Gd, cyan; Mn(II), pink; O, red; C, grey; H, white.

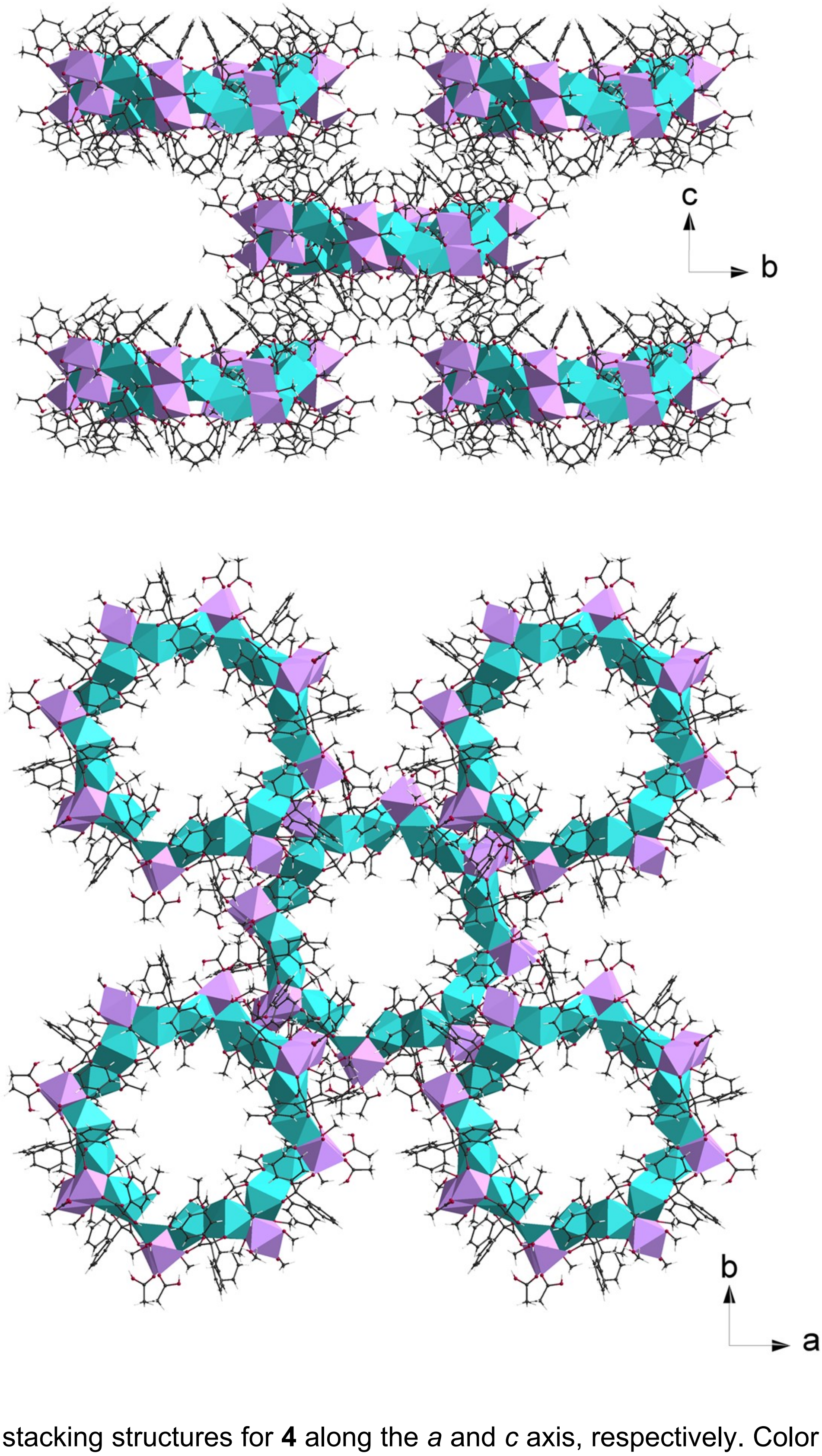

Figure S6. The stacking structures for **4** along the *a* and *c* axis, respectively. Color codes: Gd, cyan; Mn(II), pink; O, red; C, grey; H, white.

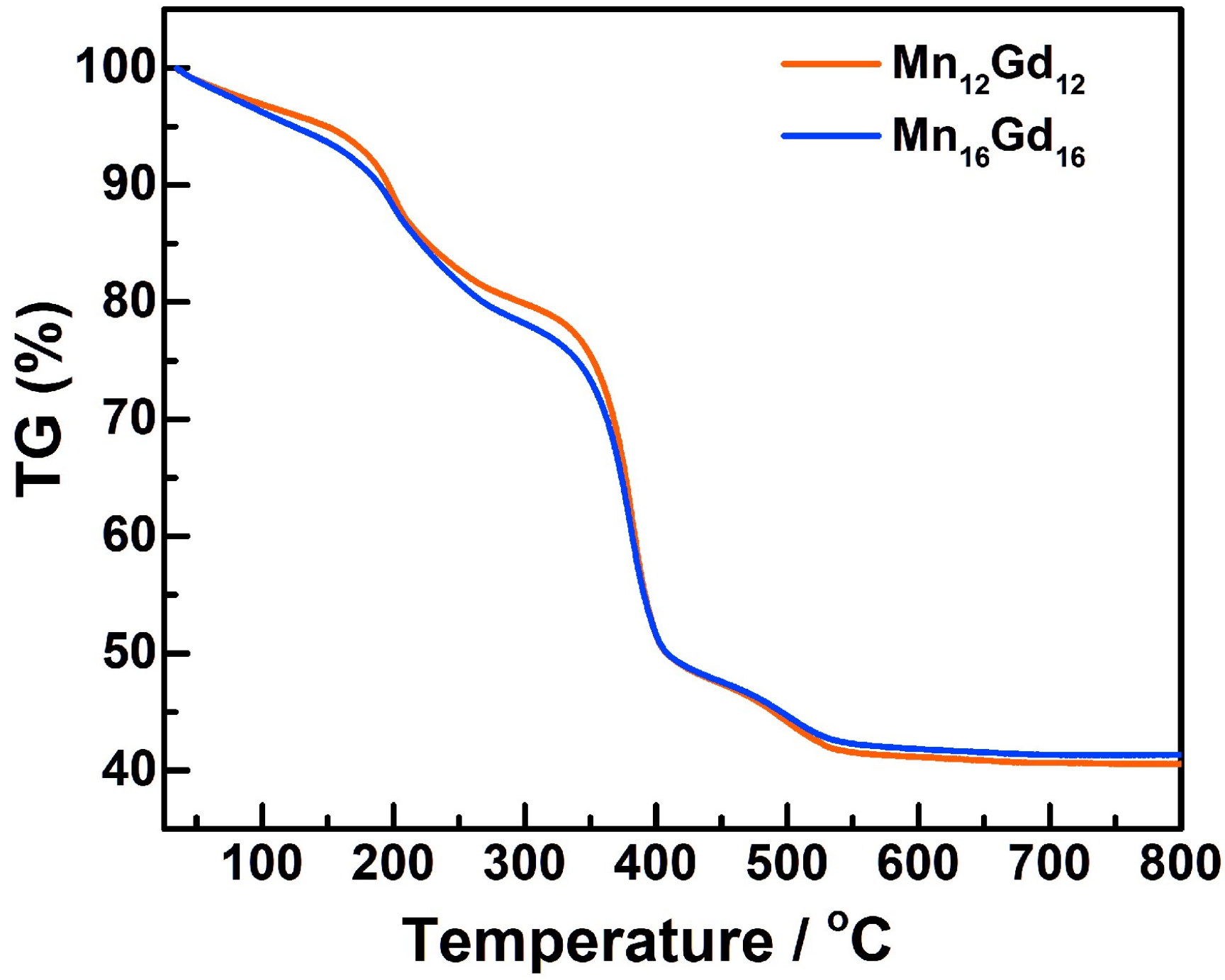

Figure S7. Thermogravimetric analysis for **1** and **4**.

The thermogravimetric (TG) curves of clusters **1** and **4** were recorded under a nitrogen atmosphere, heating from room temperature to 800 °C. For **1**, a weight loss of 6.47% is observed between room temperature and 170 °C, which is attributed to the removal of guest molecules (*calcd*. 6.50%). Upon further heating, the cluster framework begins to decompose. At 800 °C, a residual mass of 41.33% remains, corresponding to the metallic oxide residue. For **4**, the TG curve shows a weight loss of 7.88% in the temperature range of room temperature to 170 °C, consistent with the removal of guest molecules (*calcd*. 7.90%). The subsequent weight loss between 170 and 700 °C is ascribed to the decomposition and structural collapse of the framework skeleton.

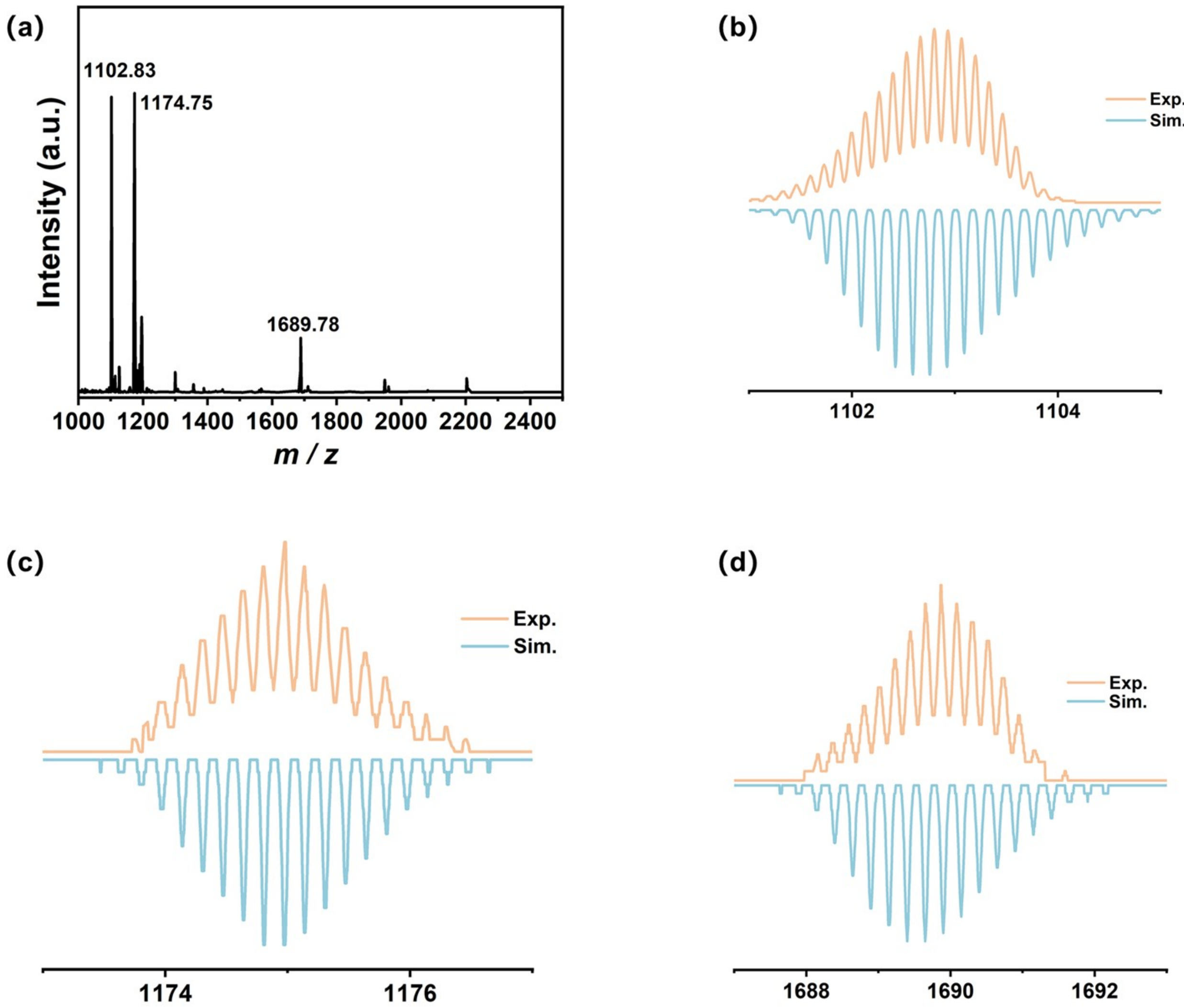

Figure S8. Positive-mode ESI-MS spectrum of **1** in dimethyl sulfoxide solution.

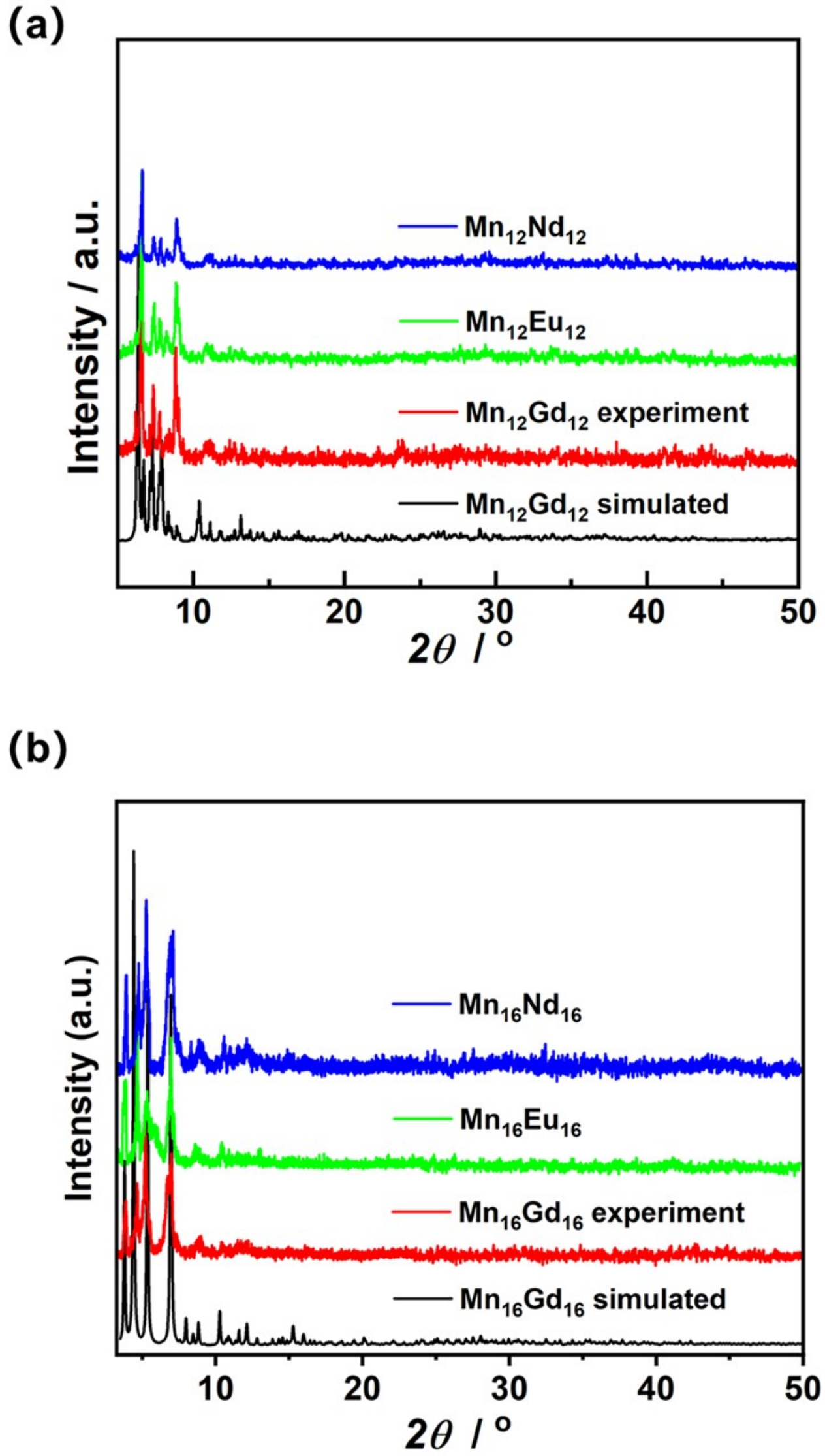

Figure S9. The PXRD patterns of **1-6**. It should be noted that a diffraction peak around 9° is present in both the experimental and simulated PXRD patterns for $Mn_{12}Ln_{12}$. The difference in relative intensity may be attributed to preferred orientation effects, variations in crystallinity, and/or partial loss of lattice solvent molecules during sample preparation and measurement.

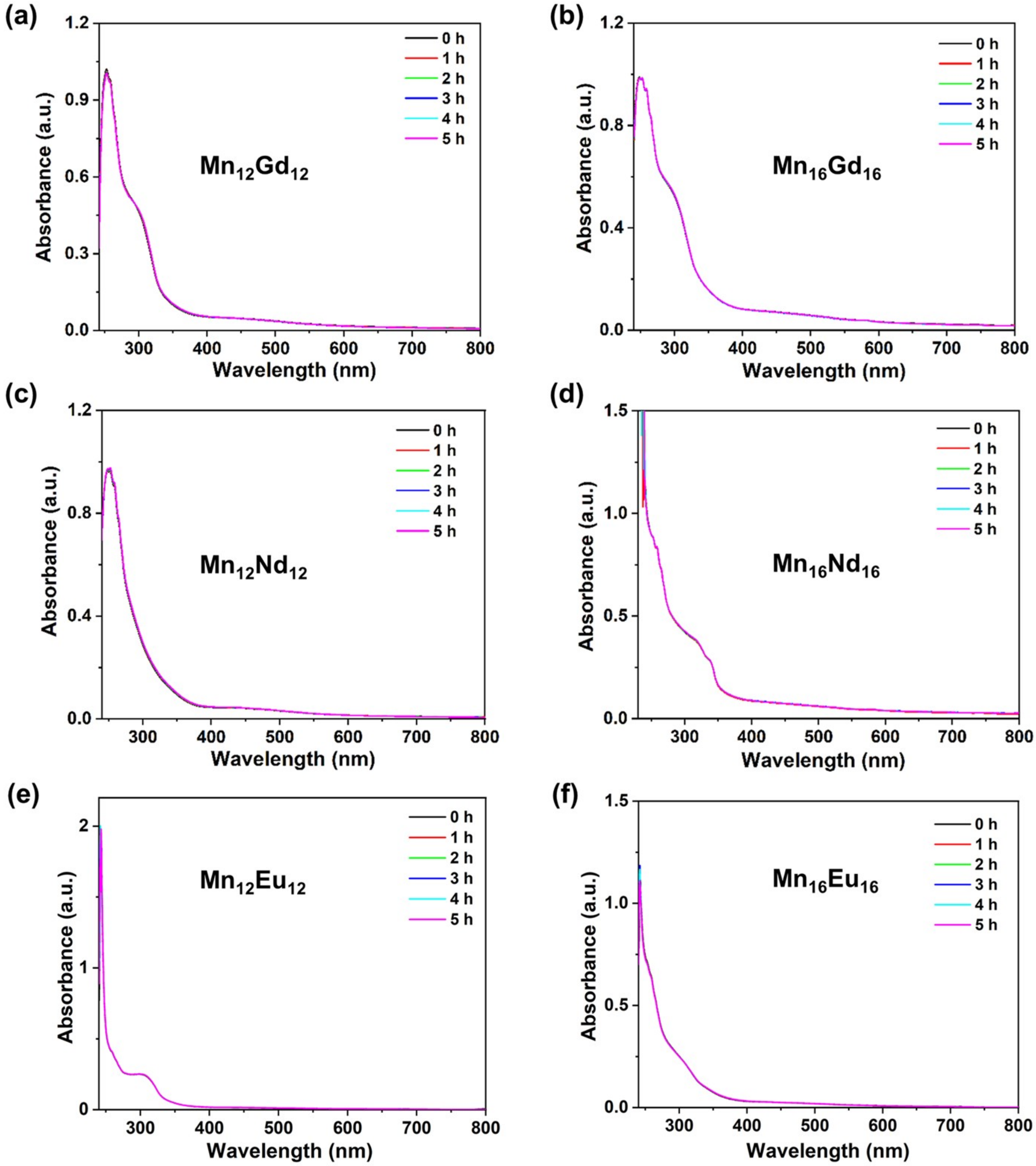

Figure S10. UV-vis spectra of 0.03 mM different catalysis in H_2O/DMSO (20 mL, *v*/*v* = 19:1) after the irradiation of 0-5 h.

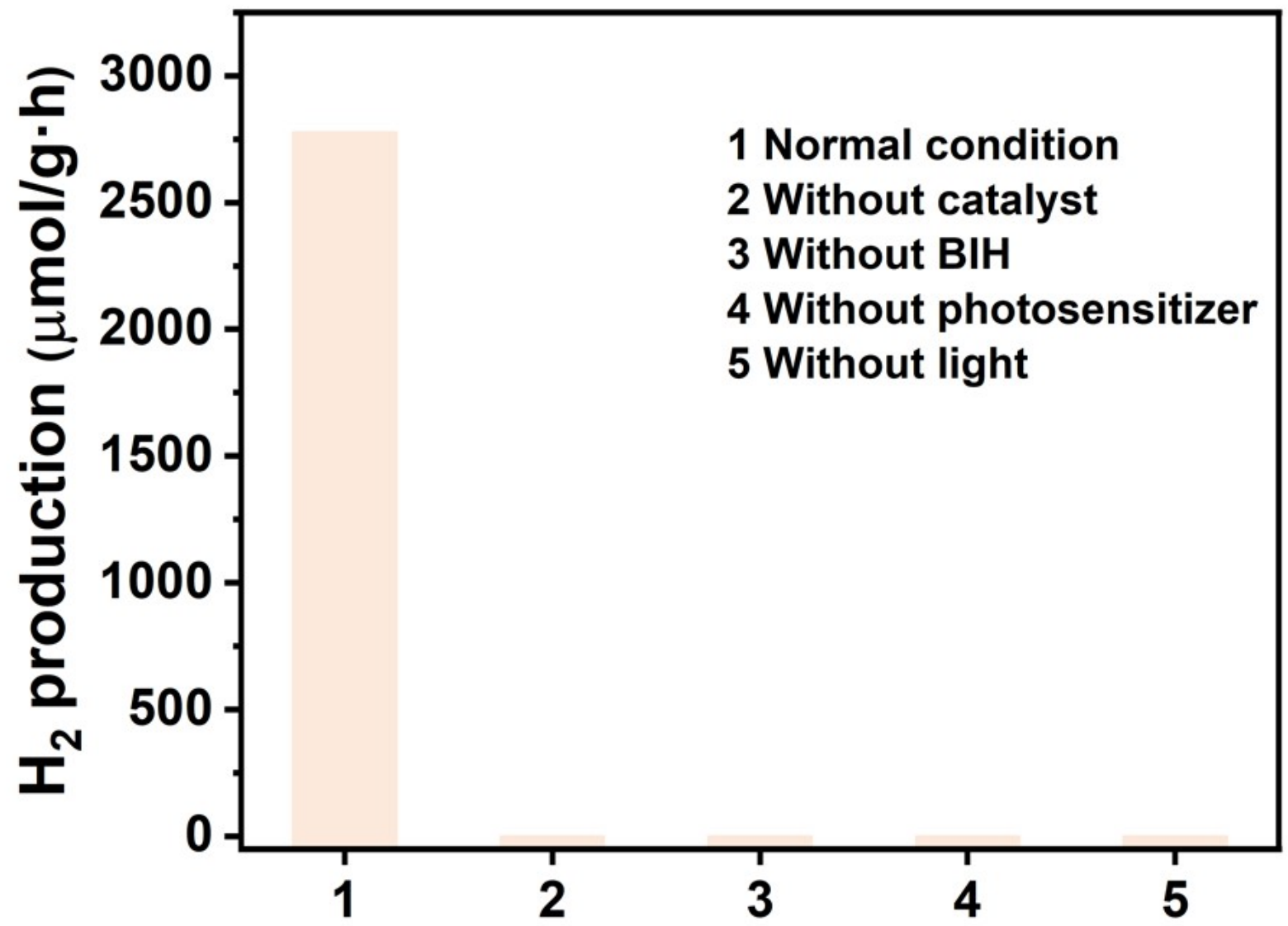

Figure S11. Photocatalytic H_2 production activity of **1** under the altered conditions.

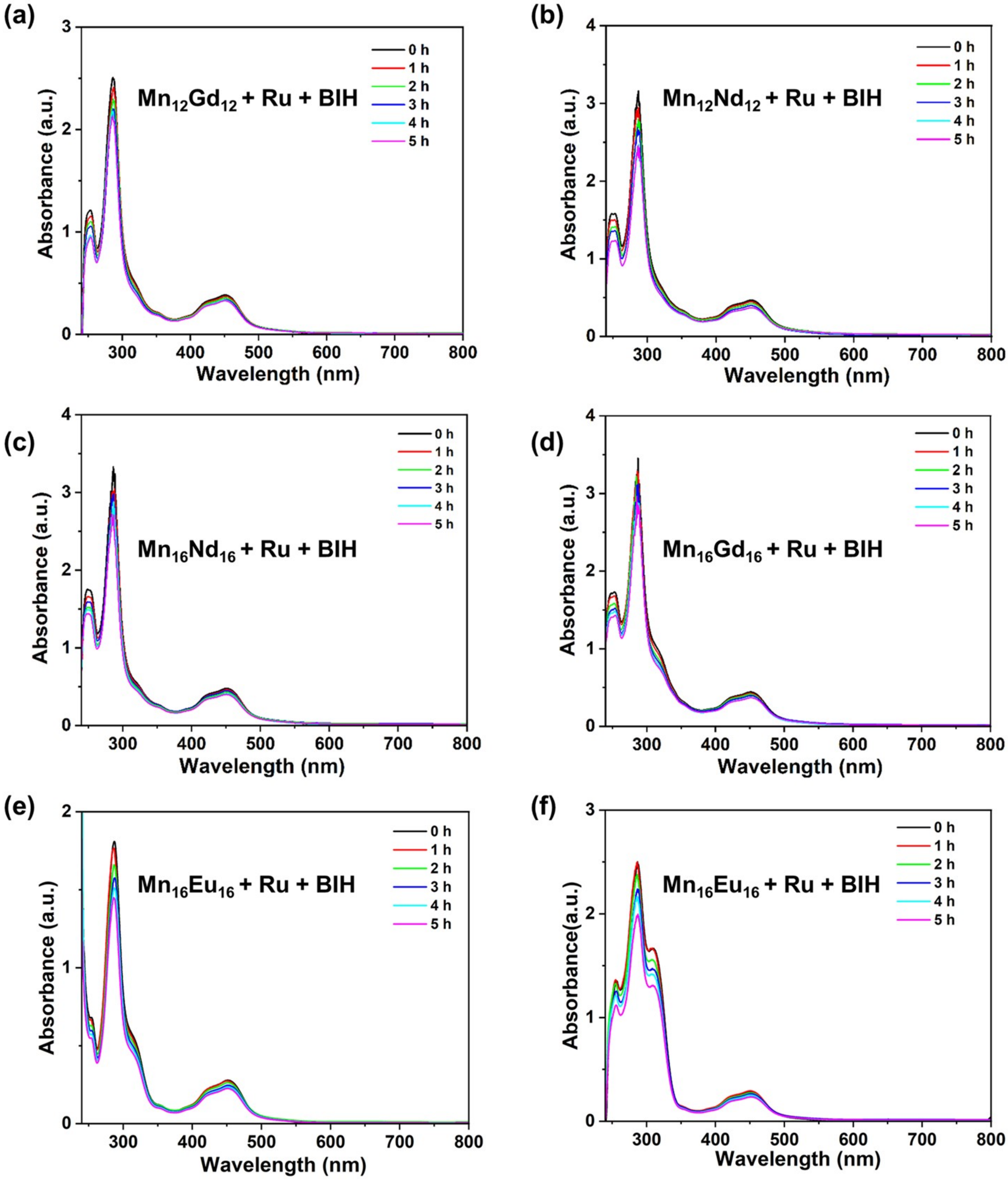

Figure S12. UV-vis spectra of 0.03 mM different catalysis, 20 mg photosensitizer $[Ru(bpy)_3]Cl_2 \cdot 6H_2O$, 40 mg BIH in H_2O/DMSO (20 mL, *v*/*v* = 19:1) after the irradiation of 0-5 h.

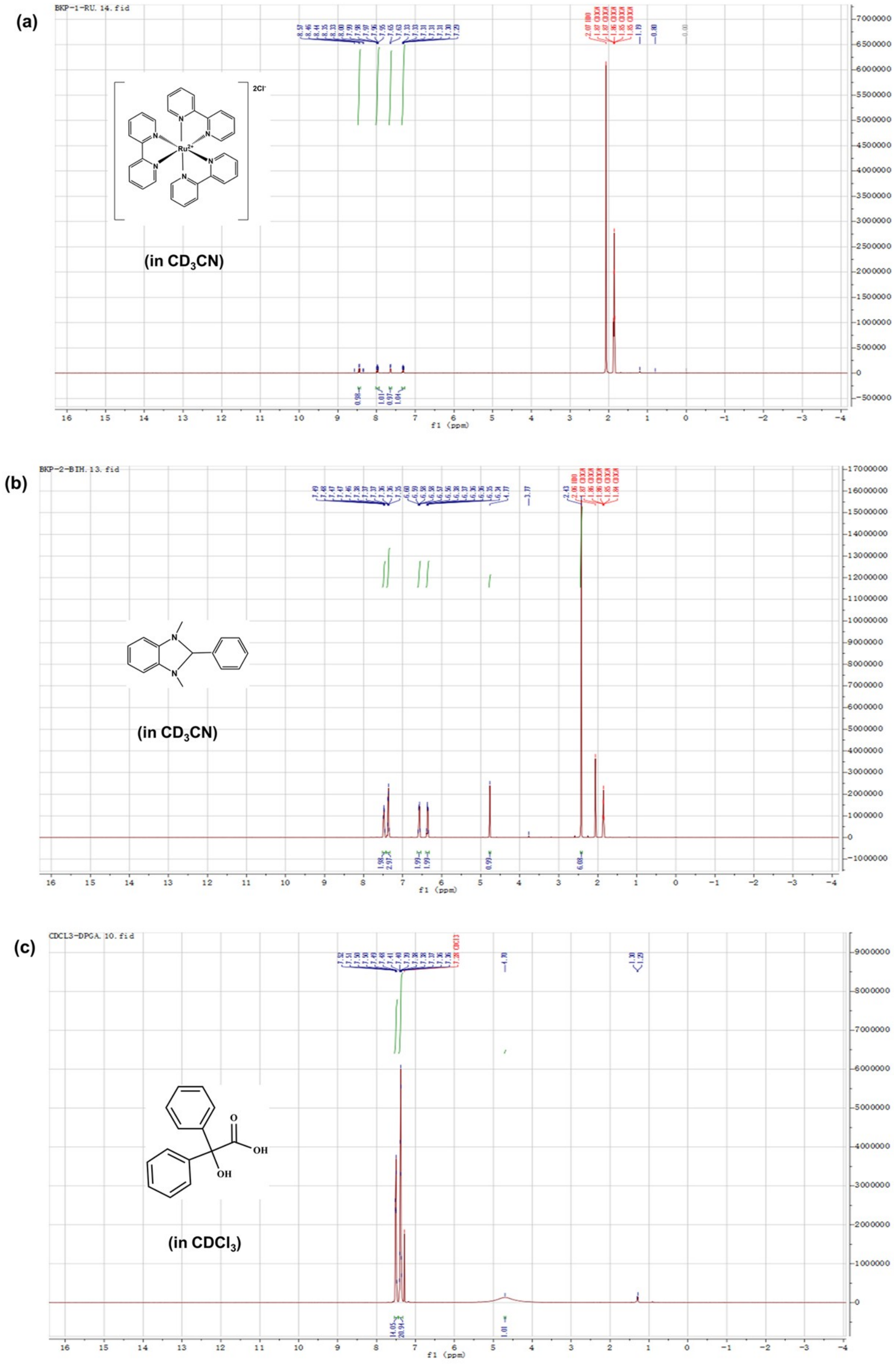

Figure S13. ^{1}H NMR spectra of $[Ru(bpy)_3]Cl_2{\cdot}6H_2O$ (a), BIH (b) and ligand H_2dpga (c).

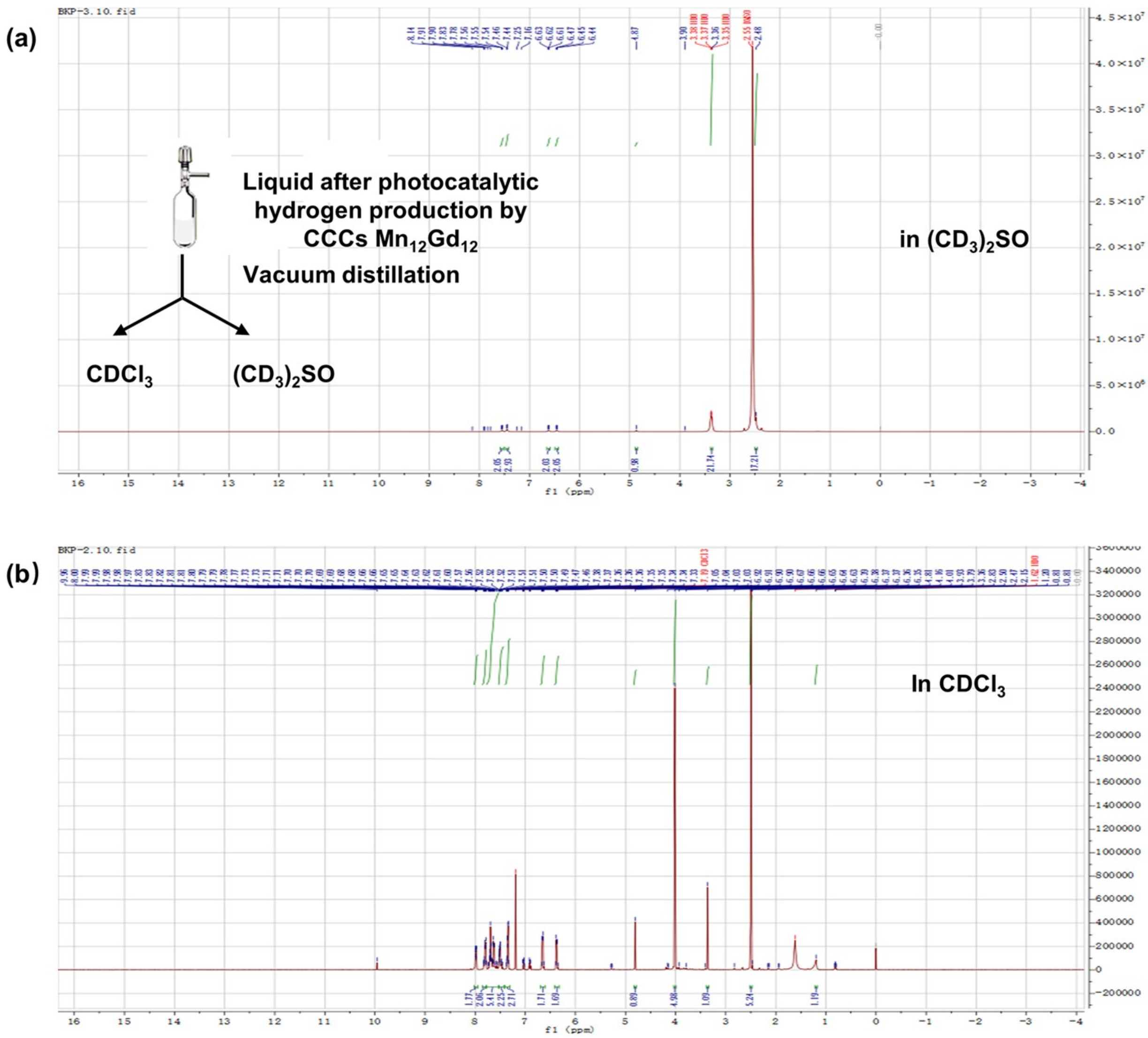

Figure S14. ^{1}H NMR spectra of **1**.

Under ultrasonic treatment, the ligand H_2dpda is soluble in $CDCl_3$. In contrast, the complex of **1** is soluble in DMSO but insoluble in $CDCl_3$. NMR experiments indicate that no distinct peak corresponding to the decomposition-generated free H_2dpga ligand was observed in the liquid products obtained from the photocatalytic hydrolysis reaction using **1**. This confirms the chemical stability of the **1** core following photocatalytic reduction.

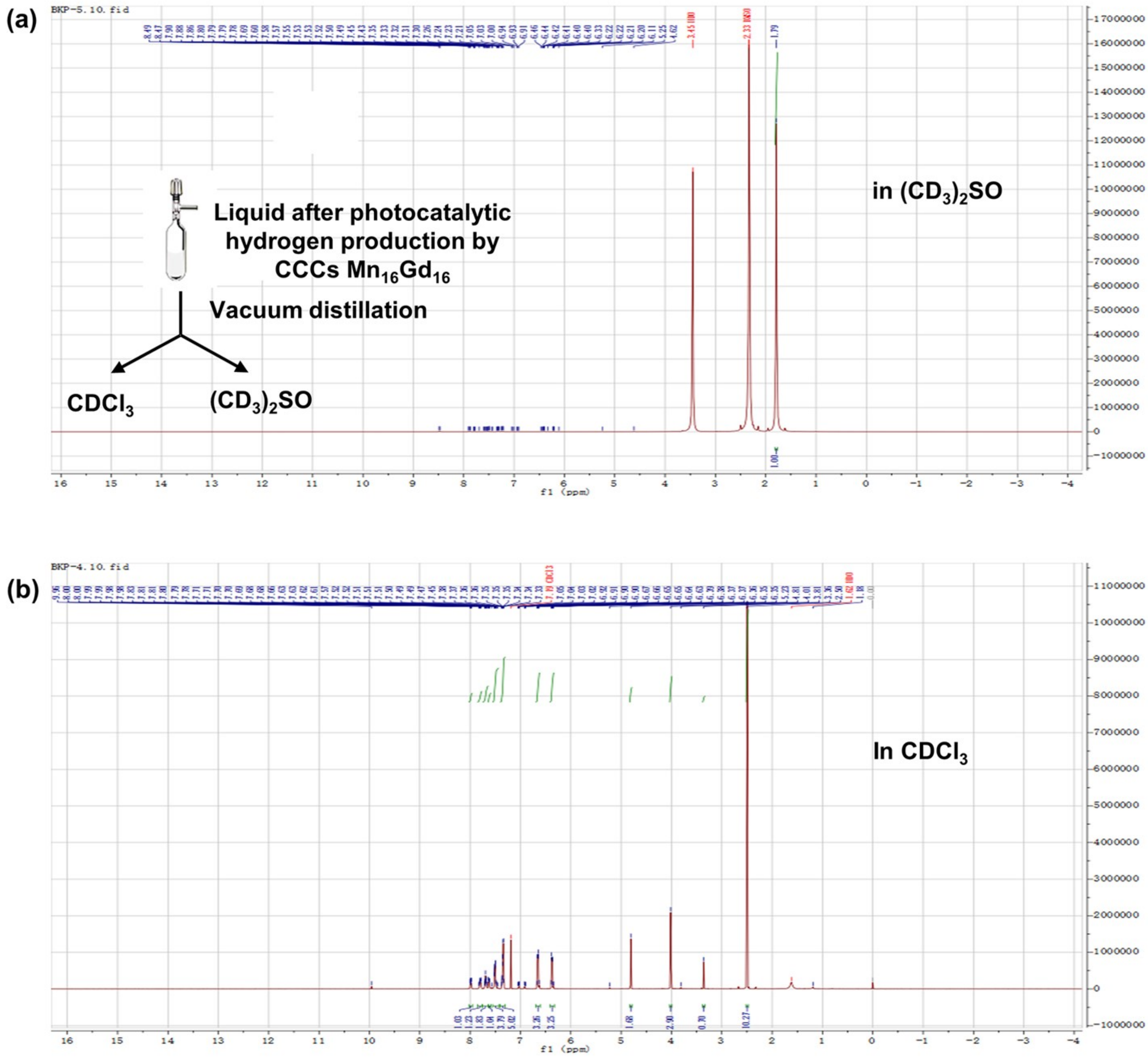

Figure S15. ^{1}H NMR spectra of **4**.

^{1}H NMR spectrum of the liquid products (a and b) obtained from the photocatalytic hydrolysis reaction using **4**. The NMR results indicate that no distinct peak corresponding to the free H_2dpga ligand generated by decomposition was observed in the liquid product obtained from the photocatalytic hydrolysis reaction using **4**. This confirms the chemical stability of the **4** core following photocatalytic reduction.

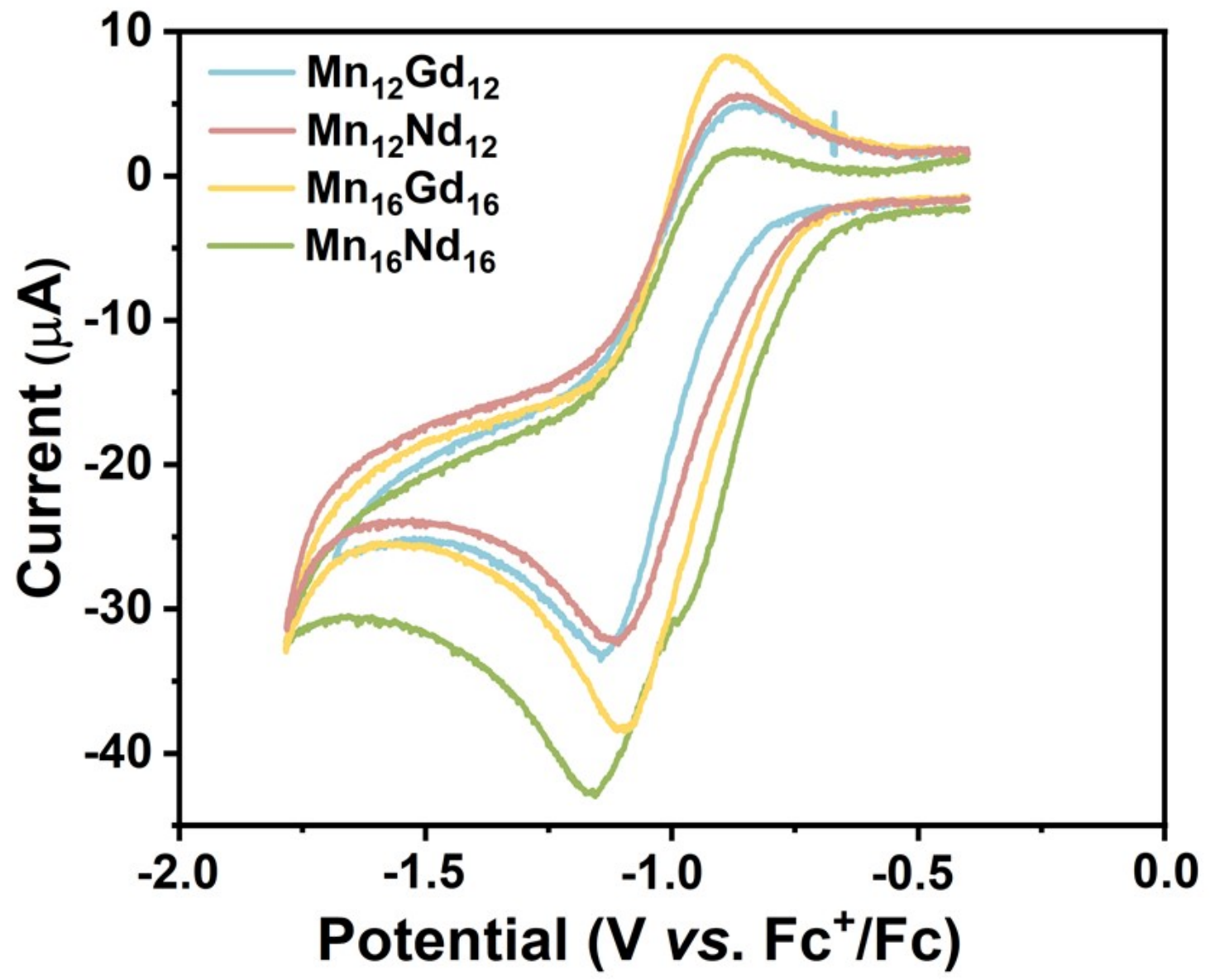

Figure S16. Cyclic voltammograms of the four clusters.

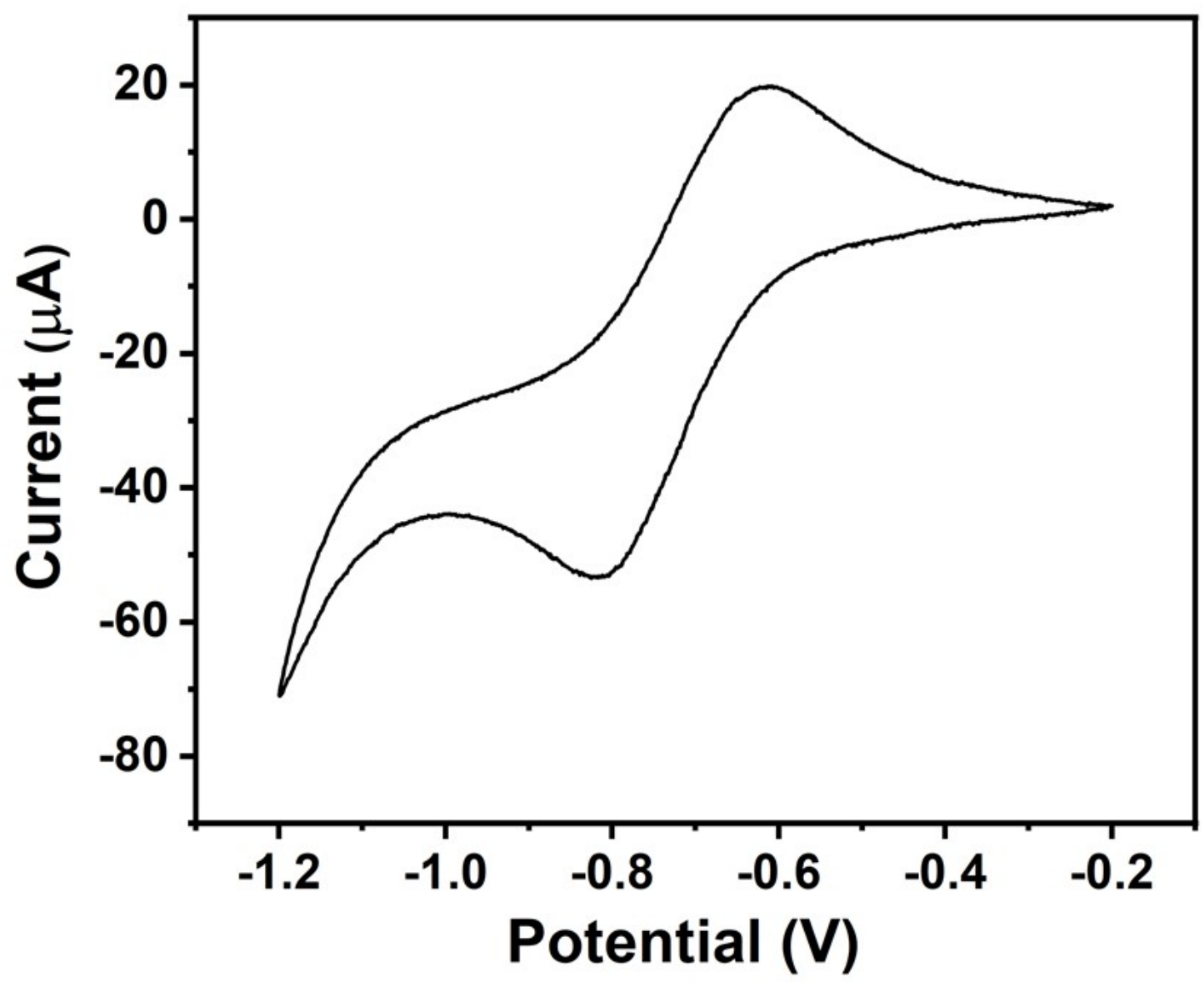

Figure S17. Potentials of Ag/AgCl are referenced to the ferrocene/ferrocenium couple. The working electrode was a 3 mm glassy carbon, the counter electrode was a platinum wire auxiliary electrode, and an Ag/AgCl was used as a reference electrode, and 2 mM ferrocene in 0.2 M Na_2SO_4 solution (H_2O/DMSO = 19:1).

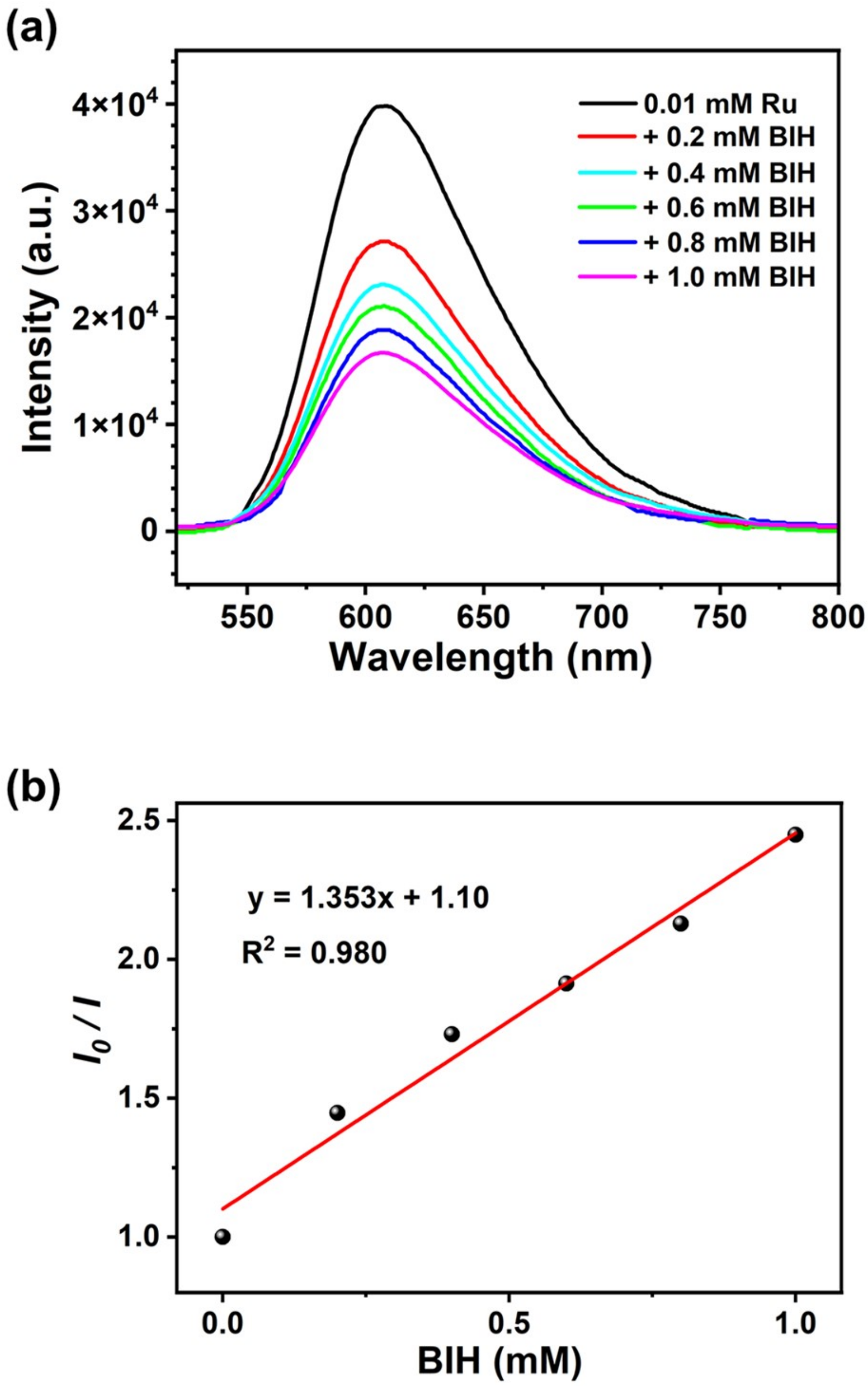

Figure S18. (a) The steady-state fluorescent spectra of $[Ru(bpy)_3]Cl_2$ (0.01 mM) upon the addition of increasing amounts of BIH (λ_{ex} = 415 nm). (b) Stern-Volmer plot of the emission quenching of $[Ru(bpy)_3]Cl_2$ solution by BIH.

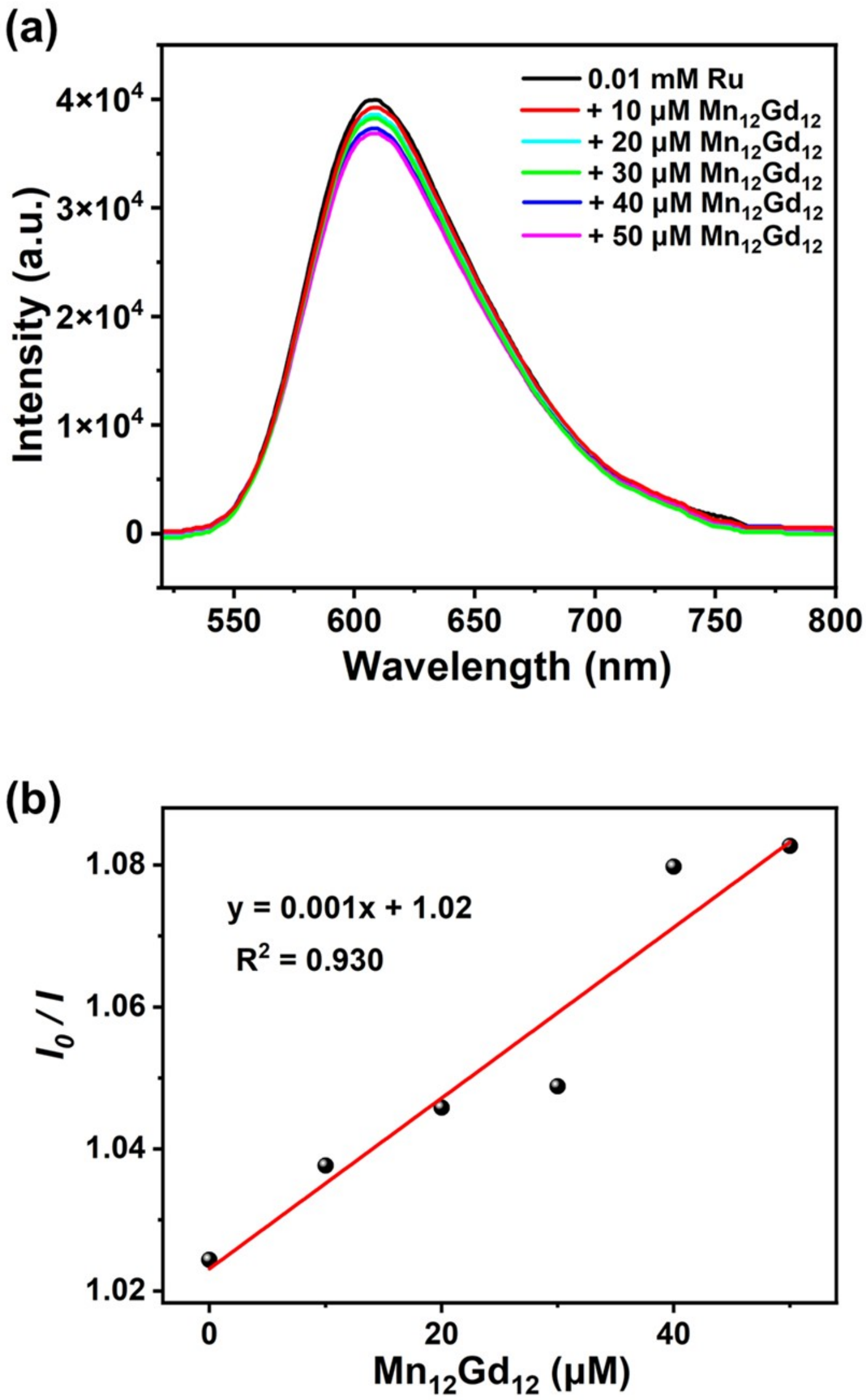

Figure S19. (a) The steady-state fluorescent spectra of $[Ru(bpy)_3]Cl_2$ (0.01 mM) upon the addition of increasing amounts of **$Mn_{12}Gd_{12}$** (λ_{ex} = 415 nm). (b) Stern-Volmer plot of the emission quenching of $[Ru(bpy)_3]Cl_2$ solution by **$Mn_{12}Gd_{12}$**.

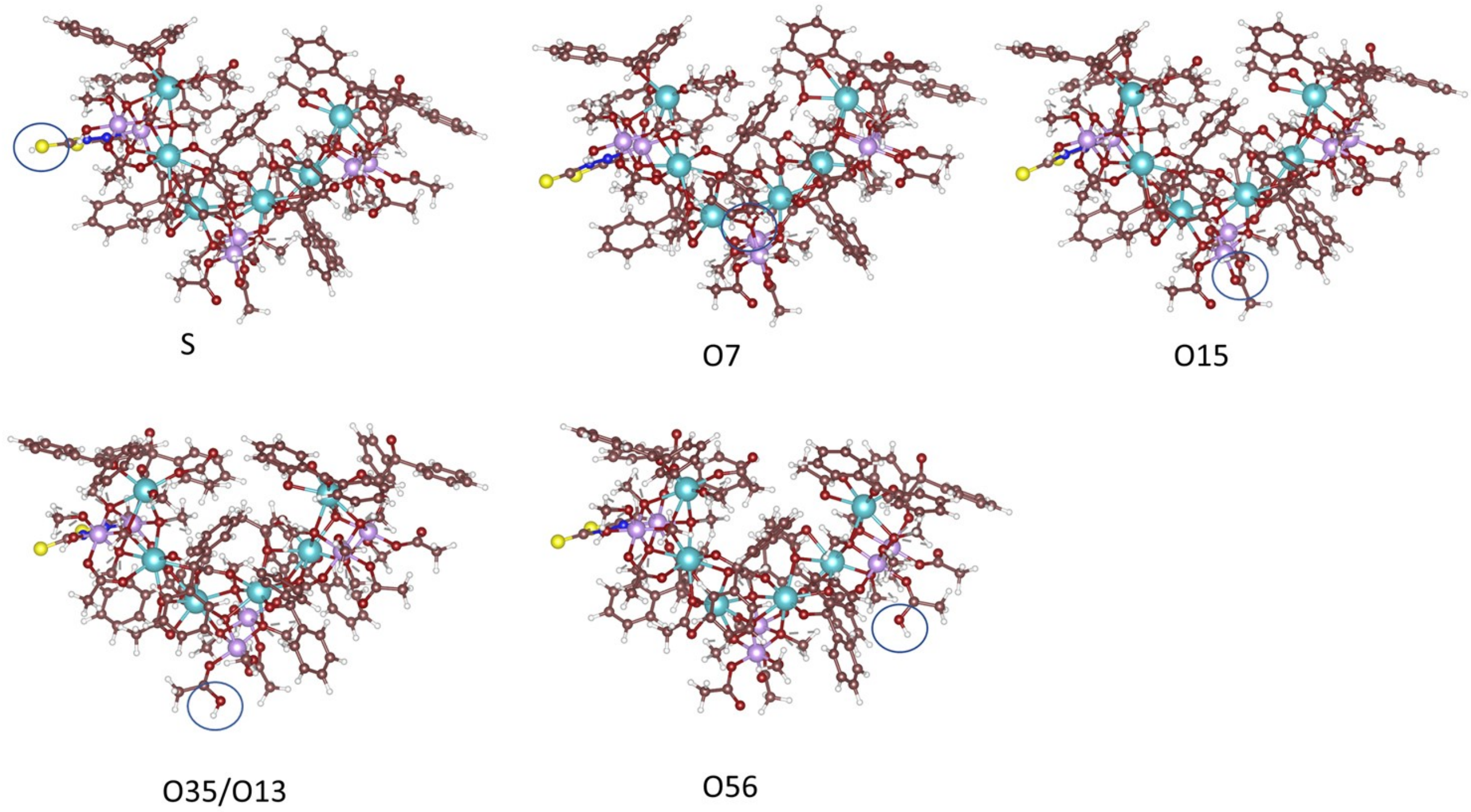

Figure S20. The different adsorption sites in cluster **1**.

(a)

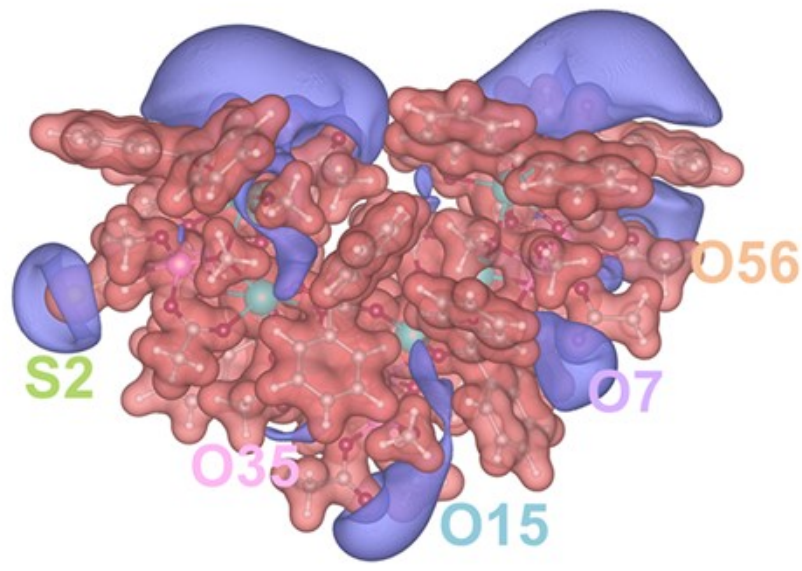

(b)

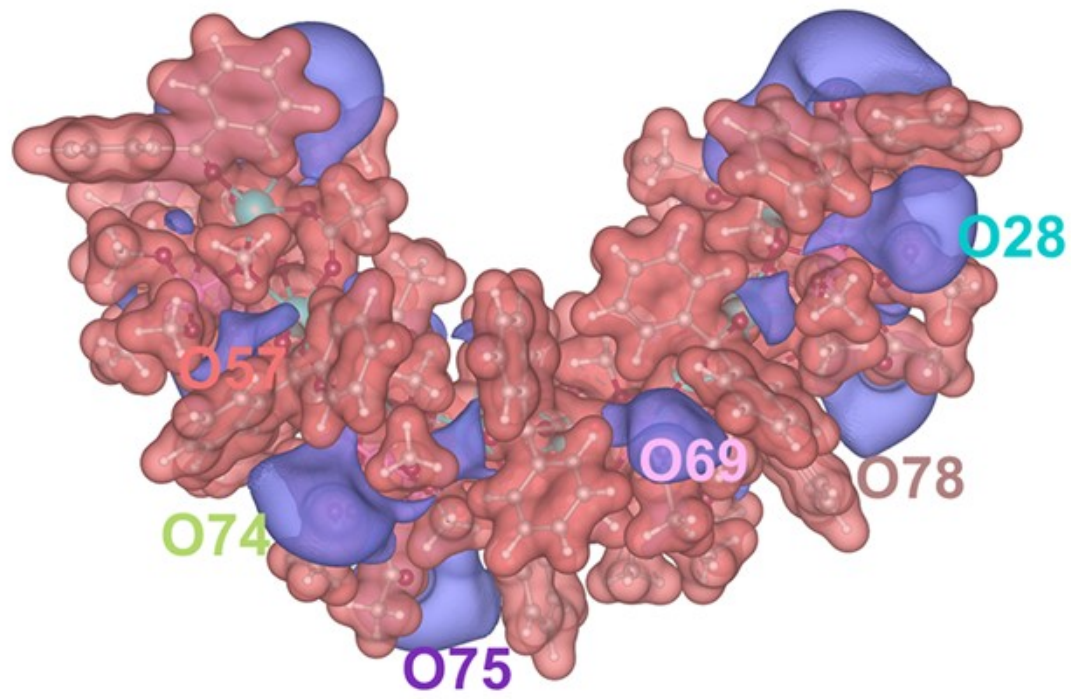

$Mn_{12}Gd_{12}$	ΔG_H	$Mn_{16}Gd_{16}$	ΔG_H
S2	-0.382 eV	O28	-0.441
O7	-0.599 eV	O57	-0.403
O15	0.501 eV	O69	-0.522
O35	-0.499 eV	O74	-0.617
O56	-0.549 eV	O75	0.420
		O78	0.596

Figure S21. Local density maps of **1** (a) and **4** (b), and Gibbs free energies at different adsorption sites.

Table S3. Comparison of the photocatalytic hydrogen production performance of several organic or inorganic catalysts systems

Photocatalysts	H_2 rate (μmol g^{-1} h^{-1})	TON	AQE (%)	Modification	Sacrificial agents	*Ref.*
$Mn_{12}Gd_{12}$	2778	112	0.47 (420 nm)	/	BIH	**This work**
Tp-Py-COF	22450	/	3.3 (420 nm)	/	ascorbic acid	6
TPCB B-COF	1029	/	79.69 (470 nm)	/	TEOA	7
Re-metallacage (4e)	1707	157	0.65	/	BIH	8
MS-c@TpPa-1	528	/	0.54 (420 nm)	/	sodium ascorbate	9
Co/Zn-Salen-COF	1378	/	/	/	ascorbic acid	10
[Co-1b]-COF	160	81	/	/	TEOA	11
Ni-Py-COF	626	/	/	/	ascorbic acid	12
Sp^2c-Py-BT COF	890	/	2.53 (420 nm)	/	/	13
MOC-16	380	635	/	/	TEOA	14
COF-TpPa-1/TiO_2	1370	/	/	/	ascorbic acid	15
$ZnIn_2S_4$@NH_2-MIL-125(Ti)	2204	/	4.30 (420 nm)	/	Na_2SO_3/Na_2S	16
$ZnIn_2S_4$/$NiSe_2$	1487	/	6.1 (420 nm)	/	TEOA	17
TpPa-1-COF/$ZnIn_2S_4$	853	/	2.08 (420 nm)	/	Na_2SO_3/Na_2S	18
$Cu_3(HHTP)_2$-MOF/Tp-Pa-1-COF	1760	/	/	/	sodium ascorbate	19
$Ni(OH)_2$/TpPa-2	1896	/	/	/	sodium ascorbate	20
ZnPor-DETH-COF 8	413	/	0.32 (450 nm)	8 wt% Pt	TEOA	21
CN/TMP	2060	/	/	Pt	TEOA	22
TAB-TFP-COF	670	/	/	3 wt% Pt	ascorbic acid	23
HBT-COF	3800	/	/	3 wt% Pt	ascorbic acid	24
CTF-1/Rgo-2	890	/	/	1 wt% Pt	TEOA	25
USTB-10	21800	/	/	3 wt% Pt	ascorbic acid	26
Pt@CTF-py-1	14960	/	/	2.51 wt% Pt	TEA	27
Pt@NH_2-UiO-66@MnO_x	1341	/	/	3.09 wt% Pt	TEOA	28
Pt-TBPyT-COF	569	/	8.91 (450 nm)	5.32 wt% Pt	sodium ascorbate	29
$NdCo_3$/PCN-c	298	/	2.0 (350 nm)	graphitic carbon nitrides	/	30
$AuAg_{24}$@UiO-66-NH_2	3600	/	0.53 (380 nm)	0.1 wt% Au	TEOA	31
Pt@UiO-99-NH_2	257	/	/	2.81 wt% Pt	TEOA	32
ReP-TiO_2/MOC-Q2	964	458	/	Re	BIH	33

Table S4. The CShM calculations of hexa-coordinate Mn(II) ions in **1**.

Refcode [a] / Com [b]	HP-6	PPY-6	OC-6	TPR-6	JPPY-6
Mn1	29.996	27.518	**0.585**	15.421	30.564
Mn2	31.300	28.546	**0.467**	15.365	31.762
Mn3	31.447	27.441	**0.481**	14.826	30.446
Mn4	32.742	28.932	**0.392**	15.735	32.391
Mn5	33.204	25.815	**1.103**	12.843	30.093
Mn6	31.888	28.153	**0.323**	15.204	32.184

[a] HP-6, hexagon; PPY-6, pentagonal pyramid; OC-6, octahedron; TPR-6, trigonal prism, JPPY-6, johnson pentagonal pyramid J2. [b] The Mn ions in each asymmetric unit of **1**.

Table S5. The CShM (Continuous Shape Measures) calculations of octa-coordinate Gd(III) ions in **1**.

Refcode [a] / Com	OP-8	HPY-8	HBPY-8	CU-8	SAPR-8	TDD-8	JGBF-8	JETBPY-8	JBTPR-8	BTPR-8	JSD-8	TT-8	ETBPY-8
Gd1	31.361	19.228	16.989	15.800	6.616	4.808	13.439	22.606	5.244	**4.570**	6.722	16.362	18.007
Gd2	32.175	20.510	16.091	13.899	4.408	**2.760**	10.996	24.272	3.305	3.040	3.786	14.488	19.659
Gd3	32.161	19.993	15.815	14.186	6.051	3.588	13.035	24.738	3.671	**2.924**	5.526	14.795	20.693
Gd4	32.369	20.441	16.398	13.879	4.355	3.176	13.440	24.472	3.795	**3.175**	5.210	14.436	19.763
Gd5	31.677	20.563	16.172	12.972	3.460	**2.724**	13.965	24.342	3.385	2.852	4.835	13.527	20.043
Gd6	32.663	21.388	14.193	10.406	3.990	**1.897**	13.728	24.833	2.986	2.523	4.234	11.087	21.563

[a] OP-8, octagon; HPY-8, heptagonal pyramid; HBPY-8, hexagonal bipyramid; CU-8, cube; SAPR-8, square antiprism; TDD-8, triangular dodecahedron; JGBF-8, johnson gyrobifastigium J26; JETBPY-8, johnson elongated triangular bipyramid J14; JBTP-8, johnson biaugmented trigonal prism J50; BTPR-8, biaugmented trigonal prism; JSD-8, snub diphenoid J84; TT-8, triakis tetrahedron; ETBPY-8, elongated trigonal bipyramid.

Table S6. The CShM calculations of hexa-coordinate Mn(II) ions in **4**.

Refcode / Com	HP-6	PPY-6	OC-6	TPR-6	JPPY-6
Mn1	31.237	28.158	**0.537**	15.197	31.509
Mn2	30.723	27.126	**0.610**	14.689	30.080
Mn3	29.982	27.707	**0.711**	14.898	30.543
Mn4	31.980	27.774	**0.340**	14.061	31.527
Mn5	29.939	27.704	**0.684**	15.186	30.250
Mn6	31.089	28.157	**0.319**	15.677	31.459
Mn7	30.311	27.013	**0.781**	14.463	29.896
Mn8	32.401	27.874	**0.565**	15.683	32.026

Table S7. The CShM calculations of octa-coordinate Gd(III) ions in **4**.

Refcode / Com	OP-8	HPY-8	HBPY-8	CU-8	SAPR-8	TDD-8	JGBF-8	JETBPY-8	JBTPR-8	BTPR-8	JSD-8	TT-8	ETBPY-8
Gd1	31.512	20.867	16.750	11.835	2.832	3.193	14.199	22.604	3.145	**2.550**	5.206	12.516	18.156
Gd2	31.678	21.021	16.853	12.259	3.151	3.156	13.438	23.483	3.176	**2.570**	5.221	12.800	18.799
Gd3	33.376	20.924	15.946	13.825	5.274	2.955	12.885	25.125	2.850	**2.345**	4.593	14.343	21.773
Gd4	33.230	21.351	16.001	14.349	4.994	3.307	13.044	25.094	2.701	**2.170**	4.946	14.888	21.364
Gd5	32.891	20.999	16.104	14.157	5.170	3.468	12.594	25.370	2.783	**2.288**	4.944	14.740	21.362
Gd6	33.432	21.259	15.995	14.071	5.007	3.272	13.176	25.325	2.778	**2.252**	5.058	14.629	21.400
Gd7	31.970	20.610	16.480	11.448	3.022	3.173	13.696	22.981	3.178	**2.480**	5.381	12.081	18.322
Gd8	31.309	20.797	16.792	12.115	2.780	3.404	14.119	22.626	3.272	**2.534**	5.584	12.667	17.975

Table S8. Selected bond distances (Å) for **1**.

Atom	Atom	Distance (Å)	Atom	Atom	Distance (Å)	Atom	Atom	Distance (Å)
Gd1	O10	2.257(3)	Gd4	O29	2.493(3)	Mn2	O44	2.219(4)
Gd1	O16	2.485(4)	Gd4	O31	2.435(4)	Mn2	O46	2.167(4)
Gd1	O18	2.506(4)	Gd4	O2	2.330(4)	Mn2	O24	2.177(4)
Gd1	O20	2.447(3)	Gd4	O35	2.417(4)	Mn2	O34	2.155(4)
Gd1	O26	2.488(3)	Gd5	O6	2.411(3)	Mn3	O6	2.272(3)
Gd1	O23[1]	2.364(3)	Gd5	O35	2.392(3)	Mn3	O2	2.193(3)
Gd1	O31	2.384(3)	Gd5	O37	2.343(4)	Mn3	O33	2.186(4)
Gd1	O51[1]	2.398(3)	Gd5	O39	2.262(3)	Mn3	O17	2.180(5)
Gd2	O18	2.422(3)	Gd5	O5	2.397(4)	Mn3	O37	2.221(4)
Gd2	O20	2.522(3)	Gd5	O41	2.528(3)	Mn3	O19	2.168(3)
Gd2	O24	2.370(3)	Gd5	O21	2.445(3)	Mn4	O7	2.138(4)
Gd2	O26	2.554(3)	Gd5	O11	2.464(3)	Mn4	O15	2.171(5)
Gd2	O30	2.276(3)	Gd6	O1	2.299(3)	Mn4	O2	2.217(4)
Gd2	O32	2.393(4)	Gd6	O4	2.331(3)	Mn4	O35	2.273(4)
Gd2	O38	2.389(3)	Gd6	O28[1]	2.390(3)	Mn4	O9	2.170(4)
Gd2	O44	2.436(3)	Gd6	O41	2.457(3)	Mn4	O37	2.174(3)
Gd3	O38	2.405(3)	Gd6	O21	2.548(3)	Mn5	O4	2.218(3)
Gd3	O40	2.365(4)	Gd6	O11	2.471(3)	Mn5	O14	2.201(4)
Gd3	O44	2.409(3)	Gd6	O23	2.400(3)	Mn5	O3	2.207(4)
Gd3	O48	2.427(4)	Gd6	O51	2.457(3)	Mn5	O49	2.189(5)
Gd3	O50	2.301(4)	Mn1	O22	2.172(4)	Mn5	O25	2.202(4)
Gd3	O52	2.490(3)	Mn1	O24	2.178(3)	Mn5	O51	2.208(3)
Gd3	O55	2.498(3)	Mn1	O38	2.275(3)	Mn6	O4	2.176(3)
Gd3	O29	2.530(3)	Mn1	O40	2.219(4)	Mn6	O12	2.173(4)
Gd4	O6	2.411(3)	Mn1	O42	2.149(3)	Mn6	O43	2.149(4)
Gd4	O52	2.473(3)	Mn1	N1	2.237(5)	Mn6	O23	2.291(3)
Gd4	O54	2.259(4)	Mn2	O36	2.232(5)	Mn6	O47	2.150(4)
Gd4	O55	2.473(3)	Mn2	O40	2.207(3)	Mn6	O3	2.189(4)

Symmetry code: [1]1-X, 1-Y, 1-Z

Table S9. Selected bond distances (Å) for **4**.

Atom	Atom	Distance (Å)	Atom	Atom	Distance (Å)	Atom	Atom	Distance (Å)	Atom	Atom	Distance (Å)

Gd1	O81	2.415(11)	Gd4	O54	2.497(10)	Gd8	O12	2.375(11)	Mn4	O37	2.165(12)
Gd1	O16[1]	2.494(11)	Gd4	O68	2.371(12)	Gd8	O36	2.260(12)	Mn4	O46	2.280(11)
Gd1	O24	2.292(12)	Gd4	O47[1]	2.446(11)	Gd8	O49[3]	2.422(12)	Mn4	O58	2.219(12)
Gd1	O26	2.450(11)	Gd4	O51	2.358(12)	Gd8	O2	2.432(12)	Mn4	O57	2.166(14)
Gd1	O28[1]	2.339(11)	Gd5	O8	2.407(11)	Gd8	O42[3]	2.510(11)	Mn5	O31	2.227(15)
Gd1	O31	2.450(12)	Gd5	O18	2.352(12)	Gd8	O46	2.368(11)	Mn5	O52	2.225(11)
Gd1	O40[1]	2.428(11)	Gd5	O22	2.467(13)	Gd8	O50	2.409(11)	Mn5	O56	2.171(12)
Gd1	O48[1]	2.353(11)	Gd5	O28	2.521(11)	Gd8	O52	2.421(11)	Mn5	O12	2.204(13)
Gd2	O4	2.399(10)	Gd5	O60	2.270(13)	Mn1	O28	2.268(11)	Mn5	O5	2.198(13)
Gd2	O20[4]	2.309(10)	Gd5	O23	2.292(10)	Mn1	O34	2.180(11)	Mn5	O58	2.180(11)
Gd2	O72	2.440(11)	Gd5	O54	2.376(10)	Mn1	O60	2.136(13)	Mn6	O4	2.260(11)
Gd2	O41	2.354(13)	Gd5	O53	2.457(12)	Mn1	O63	2.189(16)	Mn6	O30	2.141(11)
Gd2	O21	2.370(11)	Gd6	O6	2.546(11)	Mn1	O48	2.180(11)	Mn6	O62	2.221(13)
Gd2	O24	2.550(12)	Gd6	O14[2]	2.369(12)	Mn1	O55	2.157(12)	Mn6	O19[4]	2.150(14)
Gd2	O42	2.472(12)	Gd6	O16	2.344(12)	Mn2	O14	2.269(11)	Mn6	O21	2.246(12)
Gd2	O50[4]	2.500(11)	Gd6	O26[2]	2.447(11)	Mn2	O66	2.191(12)	Mn6	O59	2.177(14)
Gd3	O4	2.377(10)	Gd6	O64[2]	2.546(11)	Mn2	O68	2.225(11)	Mn7	O62	2.204(13)
Gd3	O38	2.492(12)	Gd6	O66[2]	2.452(12)	Mn2	O70	2.133(13)	Mn7	O72	2.253(13)
Gd3	O62	2.357(13)	Gd6	O47	2.505(12)	Mn2	O13	2.108(13)	Mn7	O21	2.174(12)
Gd3	O72	2.431(12)	Gd6	O40	2.270(11)	Mn2	O65	2.111(16)	Mn7	O69	2.235(17)
Gd3	O74	2.490(13)	Gd7	O38	2.426(11)	Mn3	O66	2.208(14)	Mn7	O7	2.220(13)
Gd3	O43	2.325(13)	Gd7	O74	2.412(11)	Mn3	O68	2.185(11)	Mn7	O29	2.191(15)
Gd3	O25	2.383(13)	Gd7	O39	2.369(10)	Mn3	O11	2.210(12)	Mn8	O8	2.256(11)
Gd3	O44	2.571(11)	Gd7	O44	2.326(11)	Mn3	O47[1]	2.193(12)	Mn8	O10	2.198(12)
Gd4	O1	2.296(11)	Gd7	O46	2.444(13)	Mn3	O33	2.171(15)	Mn8	O60	2.226(10)
Gd4	O14	2.402(11)	Gd7	O52	2.375(11)	Mn3	O15	2.171(15)	Mn8	O45	2.164(13)
Gd4	O18	2.549(11)	Gd7	O58	2.260(12)	Mn4	O12	2.174(11)	Mn8	O71	2.189(15)
Gd4	O22	2.542(11)	Gd7	O27	2.422(12)	Mn4	O32	2.186(14)	Mn8	O48	2.187(12)

Symmetry code: [1] +Y, 1-X, 1-Z; [2] 1-Y, +X, 1-Z; [3] -Y, +X, 2-Z; [4] +Y, -X, 2-Z

References

[1] G. Kresse, J. Furthmüller. Efficiency of *ab*-initio total energy calculations for metals and semiconductors using a plane-wave basis set. *Comput. Mater. Sci*. **1996**, *6*, 15−50.

[2] J. P. Perdew, K. Burke, M. Ernzerhof. Generalized Gradient Approximation Made Simple. *Phys. Rev. Lett.* **1996**, *77*, 3865-3868.

[3] G. Kresse, J. Furthmüller. Efficient iterative schemes for ab initio total-energy calculations using a plane-wave basis set. *Phys. Rev. B* **1996**, *54*, 11169.

[4] H. J. Monkhorst, J. D. Pack, Special points for Brillouin-zone integrations. *Phys. Rev. B* **1977**, *16*, 1746-1747.

[5] S. Grimme, J. Antony, S. Ehrlich, H. Krieg. A consistent and accurate ab initio parametrization of density functional dispersion correction (DFT-D) for the 94 elements H-Pu. *J. Chem. Phys*. **2010**, *132*, 154104.

[6] X. Du, H. Ji, Y. Xu, S. Du, Z. Feng, B. Dong, R. Wang, F. Zhang, Covalent organic framework without cocatalyst loading for efficient photocatalytic sacrificial hydrogen production from water, *Nat. Commun.* **2025**, *16*, 3024.

[7] S. Altınışık, G. Yanalak, İ. H. Patır, S. Koyuncu, Viologen-Based Covalent Organic Frameworks toward Metal-Free Highly Efficient Photocatalytic Hydrogen Evolution, *ACS Appl. Mater. Interfaces.* **2023**, *15*, 18836-18844.

[8] C. Mu, L. Zhang, G. Li, Y, Hou, H. Liu, Z. Zhang, R. Zhang, T. Gao, Y. Qian, C. Guo, G. He, M. Zhang, Isoreticular Preparation of Tetraphenylethylene-based Multicomponent Metallacages towards Light-Driven Hydrogen Production, *Angew. Chem. Int. Ed.* **2023**, *135*, e202311137.

[9] Y. Wang, P. Dong, K. Zhu, A. Zhang, J. Pan, Z. Chen, Z. Li, R. Guan, X. Xi, J. Zhang, Embedding $[Mo_3S_{13}]^{2-}$ clusters into the micropores of a covalent organic framework for enhanced stability and photocatalytic hydrogen evolution, *Chem. Eng. J.* **2022**, *446*, 136883.

[10] W. Zhou, Q.-W. Deng, H.-J. He, L. Yang, T.-Y. Liu, X. Wang, D.-Y. Zheng, Z.-B. Dai, L. Sun, C. Liu, H. Wu, Z. Li, W.-Q. Deng, Heterogenization of Salen Metal Molecular Catalysts in Covalent Organic Frameworks for Photocatalytic Hydrogen Evolution, *Angew. Chem. Int. Ed.* **2023**, *62*, e202214143.

[11] K. Gottschling, G. Savasci, H. Vignolo-González, S. Schmidt, P. Mauker, T. Banerjee, P. Rovó, C. Ochsenfeld, B. V, Lotsch, Rational Design of Covalent Cobaloxime–Covalent Organic Framework Hybrids for Enhanced Photocatalytic Hydrogen Evolution, *J. Am. Chem. Soc.* **2020**, *142*, 12146-12156.

[12] L. Sun, M. Lu, Z. Yang, Z. Yu, X. Su, Y.-Q. Lan, L. Chen, Nickel Glyoximate Based Metal–Covalent Organic Frameworks for Efficient Photocatalytic Hydrogen Evolution, *Angew. Chem. Int. Ed.* **2022**, *61*, e202204326.

[13] J. Chen, Y. Wu, W. Zhang, J. Zhang, L. Wang, M. Zhou, F. Fan, X. Wu, H. Xu, Fully Conjugated 2D sp^2 Carbon-Linked Covalent Organic Frameworks for Photocatalytic Overall Water Splitting, *Adv. Mater*. **2024**, *36*, 2305313.

[14] S. Chen, K. Li, F. Zhao, L. Zhang, M. Pan, Y.-Z. Fan, J. Guo, J. Shi, C.-Y. Su, A metal-organic cage incorporating multiple light harvesting and catalytic centres for photochemical hydrogen production, *Nat. Commun.* **2016**, *7*, 13169.

[15] H. Shen, D. Shang, L. Li, D. Li, W. Shi, Rational design of 2D/2D covalent-organic framework/TiO_2 nanosheet heterojunction with boosted photocatalytic H_2 evolution, *Appl. Surf. Sci*. **2022**, *578* 152024.

[16] H. Liu, J. Zhang, D. Ao, Construction of heterostructured $ZnIn_2S_4$@NH_2-MIL-125 (Ti) nanocomposites for visible-light-driven H_2 production, *Appl. Catal. B: Environ*. **2018**, *221*, 433-442.

[17] L. Lai, F. Xing, C. Cheng, C. Huang, Hierarchical 0D $NiSe_2$/2D $ZnIn_2S_4$ Nanosheet-Assembled Microflowers for Enhanced Photocatalytic Hydrogen Evolution, *Adv. Mater. Interfaces*. **2021**, *8* 2100052.

[18] P. Dong, T. Cheng, J.-L. Zhang, J. Jiang, L. Zhang, X. Xi, J, Zhang, Fabrication of an Organic/Inorganic Hybrid TpPa-1-COF/$ZnIn_2S_4$ S-Scheme Heterojunction for Boosted Photocatalytic Hydrogen Production, *ACS Appl. Energy Mater.* **2023**, *6*, 1103-1115.

[19] P. Xue, X. Pan, J. Huang, Y. Gao, W. Guo, J. Li, M. Tang, Z. Wang, In Situ Fabrication of Porous MOF/COF Hybrid Photocatalysts for Visible-Light-Driven Hydrogen Evolution, *ACS Appl. Mater. Interfaces*. **2021**,*13*, 59915-59924.

[20] H. Dong, X.-B. Meng, X. Zhang, H.-L. Tang, J.-W. Liu, J.-H. Wang, J.-Z. Wei, F.-M. Zhang, L.-L. Bai, X.-J. Sun, Boosting visible-light hydrogen evolution of covalent-organic frameworks by introducing Ni-based noble metal-free co-catalyst, *Chem. Eng. J.* **2020**, *379*, 122342.

[21] R. Chen, Y. Wang, Y. Ma, A. Mal, X.-Y. Gao, L. Gao, L. Qiao, X.-B. Li, L.-Z. Wu, C. Wang, Rational design of isostructural 2D porphyrin-based covalent organic frameworks for tunable photocatalytic hydrogen evolution, *Nat. Commun*. **2021**, *12*, 1354.

[22] L. Wang, R. Lian, Y. Zhang, X. Ma, J. Huang, H. She, C.Liu, Q. Wang, Rational preparation of cocoon-like g-C_3N_4/COF hybrids: Accelerated intramolecular charge delivery for photocatalytic hydrogen evolution, *Appl. Catal. B: Environ*. **2022**, *315*, 121568.

[23] X. Wu, M. Zhang, Y. Xia, C. Ru, P. Chen, H. Zhao, L. Zhou, C. Gong, J. Wu, X. Pan, Arylboron functional covalent organic frameworks for synergistic photocatalytic hydrogen evolution, *J. Mater. Chem. A* **2022**, *10*, 17691-17698.

[24] C. Lin, X. Liu, B. Yu, C. Han, L. Gong, C. Wang, Y. Gao, Y. Bian, J. Jiang, Rational Modification of Two-Dimensional Donor–Acceptor Covalent Organic Frameworks for Enhanced Visible Light Photocatalytic Activity, *ACS Appl. Mater. Interfaces* **2021**, *13*, 27041-27048.

[25] Z. Tan, P. Zhang, Q. Chen, S. Fang, G. Huang, J. Bi, L. Wu, Visible-light-driven photocatalyst based upon metal-free covalent triazine-based frameworks for enhanced hydrogen production, *Catal. Sci. Technol.* **2021**, *11*, 1874-1880.

[26] W. Li, X. Ding, B. Yu, H. Wang, Z. Gao, X. Wang, X. Liu, K. Wang, J. Jiang, Tuning Molecular Chromophores of Isoreticular Covalent Organic Frameworks for Visible Light-Induced Hydrogen Generation, *Adv. Funct. Mater*. **2022**, *32*, 2207394,

[27] C. Li, L. Guan, J. Zhang, C. Cheng, Z. Guo, Z. Tian, L.-M. Yang, S. Jin, Regulating Pt-covalent triazine framework Schottky junctions by using tailor-made nitrogen sites towards efficient photocatalysis, *J. Mater. Chem. A* **2024**, *12*, 13876-13881.

[28] J. Zhang, T. Bai, H. Huang, M.-H. Yu, X. Fan, Z. Chang, X.-H. Bu, Metal-Organic-Framework-Based Photocatalysts Optimized by Spatially Separated Cocatalysts for Overall Water Splitting, *Adv. Mater.* **2020**, *32*, 2004747.

[29] T.-T. Sun, H. Zhang, Y. Wang, N.-N. Zhang, L.-H. Shao, H. Dong, F.-M. Zhang, Covalent Organic Frameworks Anchoring Single-Atom Pt for Three-Phase Interface-Assisted Photocatalytic Overall Water Splitting, *Angew. Chem. Int. Ed.* **2025**, *64*, e202515397.

[30] R. Chen, G.-L. Zhuang, Z.-Y. Wang, Y.-J. Gao, Z. Li, C. Wang, Y. Zhou, M.-H. Du, S. Zeng, L.-S. Long, X.-J. Kong, L.-S. Zheng, Integration of bio-inspired lanthanide-transition metal cluster and P-doped carbon nitride for efficient photocatalytic overall water splitting, *Natl Sci Rev.* **2021**, *8*, nwaa234.

[31] H. Wang, X. Zhang, W. Zhang, M. Zhou, H.-L. Jiang, Heteroatom-Doped Ag_{25} Nanoclusters Encapsulated in Metal-Organic Frameworks for Photocatalytic Hydrogen Production, *Angew. Chem. Int. Ed.* **2024**, *63*, e202401443.

[32] J.-D. Xiao, Q. Shang, Y. Xiong, Q. Zhang, Y. Luo, S.-H. Yu, H.-L. Jiang, Boosting Photocatalytic Hydrogen Production of a Metal-Organic Framework Decorated with Platinum Nanoparticles: The Platinum Location Matters, *Angew. Chem. Int. Ed.* **2016**, *55*, 9389-9393.

[33] S. Qin, Y. Lei, J.-F. Huang, C.-Y. Lv, X. Li, P.-Y. Su, J.-M. Liu, Controllable Visible-Light-Driven Syngas Evolution by a Ternary Titania Hybrid Sacrificial System with a Photosensitive Metal-Organic Pd^{II} Cage and ReI Catalyst, *ACS Sustainable Chem. Eng*. **2022**, *10*, 8254-8264.
